# Actions to decarbonize English schools: a whole life carbon stock assessment

**DOI:** 10.1007/s44498-026-00027-x

**Published:** 2026-02-26

**Authors:** Danielle Abbey, Hadi Arbabi, Danielle Densley Tingley

**Affiliations:** School of Mechanical, Aerospace, & Civil Engineering, University of Sheffield, Sheffield , UK

**Keywords:** Industrial ecology, Whole life carbon, Retrofit, Carbon budget, Decarbonisation, Pathways, Building material

## Abstract

In this paper we develop a whole life carbon stock model to assess the impacts of retrofit in cool-temperate climates. The developed model can explore different retrofit deployment rates to limit emissions of the existing building stock, and be used to develop different pathways which could meet carbon budgets for varying future scenarios. We illustrate the application of the model to the English school stock, comprising approximately 20,000 schools. In applying the model, we find that an estimated school carbon budget is only met with a significant increase in retrofit rates from current ambitions, alongside significant decarbonization in electricity and material sectors. When retrofit modeling is applied at scale, the aggregated impacts of material consumption are highlighted, and the most stringent carbon budget cannot be met when a whole life carbon approach is taken. Heat pumps and active ventilation systems pose a significant carbon hot-spot due to the use of carbon-intensive materials and regular replacement rates. These systems would therefore benefit from research and development to reduce their impacts. The developed model also allows for a stock level comparison of retrofit to the alternative of demolition and new construction (to different future standards). We show that retrofit provides the lowest whole life carbon solution over the 25 year study period for all buildings, compared to proposed ambitious UK whole life carbon targets.

**Supplementary Information:**

The online version contains supplementary material available at 10.1007/s44498-026-00027-x.

## Introduction

A global reduction of at least 64% of greenhouse gas emissions is required by 2050 to meet the 1.5–2 °C levels of warming (IPCC, 2023). 2 °C is seen as the critical threshold for escalating threats to human life (Park et al. 2023) with a finite carbon budget left to limit global warming impacts. With the built environment accounting for over one third of current emissions (Center and for Ecosystems + Architecture, 2023), large reductions in this sector are required to meet targets.

Over 80% of the existing European stock is predicted to still be standing by 2050 (Hollyman & Dokin, 2024; Renovation, 2020). With 97% of this stock requiring energy efficiency upgrades (BPIE, 2017), retrofit is vital for reducing built environment emissions. Undertaking at scale retrofit requires an increase in material consumption that carries significant emissions. A Canadian case study shows that life-cycle carbon improvements from retrofit decrease from 98% to 75% once embodied emissions are accounted for Grossi et al. (2024). Li et al. (2022) also show that the most stringent English residential carbon budget can only be met through retrofit of the entire stock with low carbon insulation materials Li et al. (2022). Whole life assessments at scale are highly useful from a policy perspective, allowing quantification of what level of intervention is needed to meet key climate targets.

Mass retrofit and decarbonization of electricity supply are crucial (Berrill et al., 2022; Ghose et al., 2020; Göswein et al., 2021; Hegarty & Kinnane, 2023; Verellen & Allacker, 2022). However, retrofit is not the only solution for improving operational efficiency of the built environment. One alternative solution would be demolition and replacement with a more efficient building than the original construction. Embodied impacts of this would be high, as current global embodied carbon benchmarks for new-builds (600–750$kgCO_2e/m^2$) are significantly larger than retrofit (20–140$kgCO_2e/m^2$) (Bienert et al., 2023). However, new buildings, while still subject to existing site constraints such as urban density, are not constrained by the features of the original building, eg, form and orientation, which are shown to impact the effectiveness of different refurbishment measures ( La Fleur et al., 2019; Pomponi et al., 2015). It is also less complex within new constructions to reach better energy efficiency standards, eg, higher airtightness levels (Gillott et al., 2016).

Whole life studies of new construction versus retrofit report conflicting results. Multiple publications show retrofit has lower environmental impacts (Berg & Fuglseth, 2018; Ferriss, 2021; Huuhka et al., 2023; Itard & Klunder, 2007; Nydahl et al., 2022; Peng et al., 2021; Pullen, 2010; Säynäjoki et al., 2012; Schwartz et al., 2022; Storck et al., 2023) with others finding new construction preferable (Atmaca et al., 2021; Dong et al., 2005; Dominguez et al., 2024; Hawkins et al., 2017a, b; Feng et al., 2020; Palacios et al., 2019; ). While these studies cover similar geographies across Europe, Australia, and north America, due to variations between individual case studies and their system boundaries (Schwartz et al., 2018), it is hard to directly compare these studies.

### Understanding the English school stock

School buildings in the UK account for approximately 2% of total national annual emissions (Energy and Industrial Strategy, 2020). These are typically old and inefficient with over half of nearly 22,000 schools in England built before building regulations were first introduced to ensure decent levels of energy efficiency (Department, 2021; Jentsch, 2013). Retrofit of schools is directly driven by local governments (Bevan et al., 2020) with explicit social goals (IET, 2020), including local decarbonization ambitions but also reductions of fuel poverty and excess winter deaths. However, there is a need to understand the impact of retrofit on the English school stock from a whole life perspective, and quantify the embodied impacts of mass intervention for a range of retrofit scenarios and measures. This paper takes a holistic approach, assessing the whole life carbon impact of retrofit at scale, modeling different retrofit scenarios as well as different levels of electricity and material decarbonization, and conducts comparisons to different new construction standards. This is indicative of many similar buildings within cool-temperate, heating dominated climates, eg, Canada and Northern Europe with old non-residential stock (NRC, 2012) with similar energy intensity levels (BPIE, 2011) heavily reliant on fossil-fuel heating systems (Abdel-Salam et al., 2022; Econometrics, 2023).

To meet net zero targets by 2050, the UK government has set an emissions cap in the form of several 5-year carbon budgets (CCC, 2020). This limits total emissions by the UK to keep in line with the Paris Agreement. A more stringent interpretation of this value is provided by the Tyndall Centre assigning developed countries a smaller proportion of the global budget (Kuriakose et al., 2022; Li et al., 2022). This paper uses both budgets to ask:

- Can allocated budgets be met through retrofit of the existing stock?
- What are the barriers from a whole life perspective to meeting targets?
- And, what are realistic renovation rates to meet them?

These are answered through development of a method which can model the entire school stock, here incorporating individual building-level information on form and geometry as well as the influence of archetype-based fabric characteristics on whole life carbon decision making.

## Methodology

**Fig. 1:**
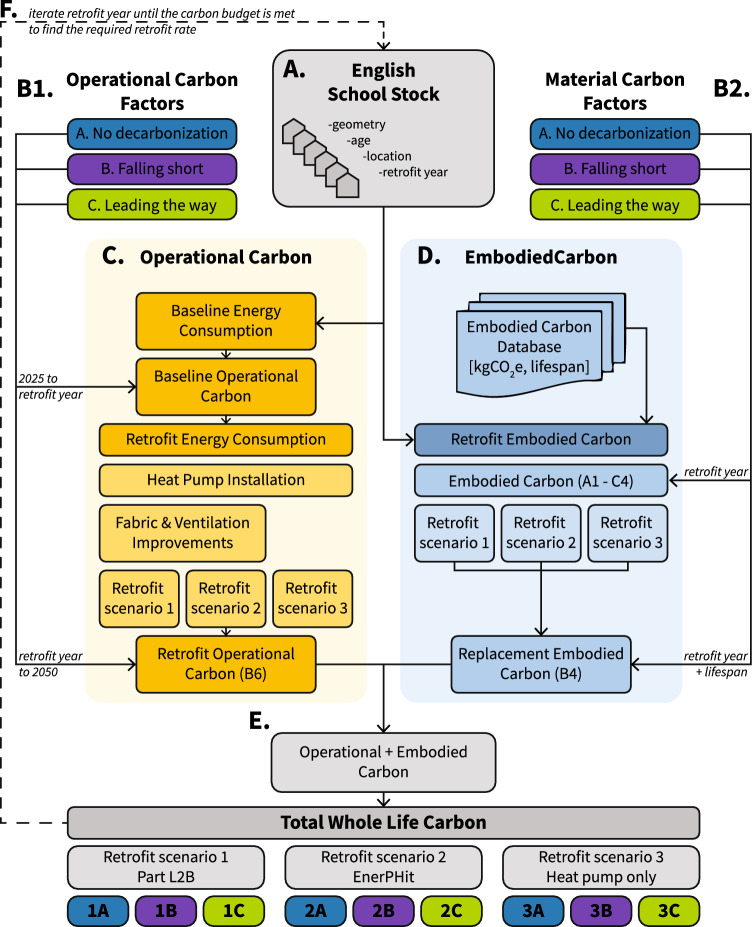
Flow chart showing whole life carbon retrofit modeling of the English school stock. All retrofit scenarios involve replacement of existing systems with a heat pump but vary in fabric efficiency levels. Building geometry, age, and location are used to calculated total operational carbon both before and after retrofit depending on the year retrofit is modeled to occur. Operational carbon is then added to the modeled embodied carbon of retrofit installations. Three different decarbonization pathways are modeled for each retrofit scenario changing annual operational carbon emissions of electricity as well as the total embodied carbon of retrofit when it is installed

This section outlines the developed methodology for whole life carbon modeling of naturally ventilated buildings in a cool-temperate climate, Fig. 1. Our case study focuses on all 20,109 English primary/secondary schools and colleges which are over 90% of the total stock (Department, 2021). The following sections outline the key methodological stages, beginning with data collection, Fig. 1A.

A comprehensive methodology including a database of all embodied and operational carbon factors is provided in Supplementary Information SI1.

### Data collection and defining building geometry

Building geometry, age and location are collected for every school in England to allow for energy and whole life carbon modeling at scale. A national database is used to obtain location of every school (Department, 2024b). All educational buildings within a defined search radius of these coordinates are extracted to find relevant schools (Department, 2024b; Digimap, 2025). Geometry is essential for estimating area through which heat is lost and age is used to define model thermal efficiency. They are used to define the baseline operational performance after (Abbey et al., 2025; Bull et al., 2014), see Supplementary Information SI1.

### Modeled scenarios

Typical occupancy data for each type of school is used to quantify key values such as internal temperature, total internal heat gains and typical hot water usage (Burgess & Rose, 2021; CIBSE 2016, 2021; Department, 2014, 2015). We acknowledged that occupancy will vary between schools and that after retrofit, due to a higher building thermal efficiency, energy usage may be increased by occupants without additional costs, an occurrence called the rebound effect (Aydin et al., 2017). Detailed assumptions for modeled scenarios can be found in Sects. 1.2, 1.3, and 1.5 of the Supplementary Information SI1 and a sensitivity analysis for different occupancy patterns in Supplementary Information SI2.

#### Defining retrofit scenarios

Three typical retrofit scenarios are modeled to understand the impact of different refurbishment measures. These are PartL2B, EnerPHit, and heat pump only. Part L2B follows UK’s building regulation for non-residential retrofit focusing on improvements to the building fabric (Housing and Communities, 2023). EnerPHit, the retrofit equivalent to PassivHaus, requires stricter levels of air-tightness and fabric efficiency. For this reason mechanical ventilation and heat recovery (MVHR) is advised to ensure safe levels of ventilation (Passivhaus, 2016). As neither PartL2B nor EnerPHit define a specific heating or hot water system, it is assumed that all in-situ fossil fuel systems are replaced with an air-to-water heat pump with operational carbon emissions estimated as a function of grid decarbonization pathways. Medium temperature heat pumps are modeled with no change to emitter size, due to the improvements to fabric efficiency (Lämmle et al., 2022).

#### Decarbonization scenarios

Three scenarios are used to analyze the impact of primary energy decarbonization (NESO, 2023), Figure 1B. These are titled no decarbonization, falling short, and leading the way, and model different levels of electricity and material decarbonization. The chosen scenarios aim to provide worst, best and typical predictions for $c_{e}$ [$kgCO_2e/kWh$] and are shown in Table 1. Material decarbonization for 3 key retrofit materials, ie, steel, glass, and insulation are also considered, although harder to predict as it relies on many factors, including the implementation of low carbon strategies which differ widely between different materials (WSP, & DNV GL, 2015). Each retrofit element will follow the decarbonization pathway of the material which takes up the majority of that element’s total mass. For example, EPDs show that steel and iron take up 44–78% of heat pumps’ and 66–80% of MVHRs’ total mass. Therefore, mechanical, electrical and plumbing system (MEP), except for refrigerants, will decarbonize following the steel and iron pathway (WSP, & DNV GL, 2015). Additionally, we consider a comparison of refrigerants - the leading the way scenario uses R774 refrigerant, which is a low carbon alternative to often modelled R513A (Hamot et al., 2020).

**Table 1:** Material and fuel decarbonization scenarios–for gas use, a constant value of $c_{g}=0.18kgCO_2e/kWh$ is used (Department for Energy Security and Net Zero 2024)

	Electricity decarbonization	Material decarbonization
No decarbonization	Constant emissions from electricity, using $c_{e_{2025}} = 0.18 kgCO_2e/kWh$ (NESO, 2023)	No material decarbonization occurs between 2025–2050
Falling short	Follows the UK electric grid’s falling short model (NESO, 2023), excluding the impacts of carbon capture and storage	Material decarbonization follows the UK government’s industrial decarbonization business as usual pathway for all 3 key materials (WSP & DNV GL, 2015)
Leading the way	The most optimistic model provided by the UK national grid and includes carbon capture and storage. (NESO, 2023)	Material decarbonization follows the UK government’s industrial decarbonization max technical pathway for all 3 key materials (WSP & DNV GL, 2015)

### Whole life carbon modeling

For each school the total operational carbon, post- and pre-retrofit, is calculated along with total embodied carbon for each retrofit scenario.We consider the 25 years leading to 2050 for whole life carbon modeling as this aligns with both the UK, and wider global, net zero decarbonization targets by this date (CCC, 2020).

#### Operational carbon

Energy consumption, stage B6 of the life-cycle assessment as defined by BS EN 15643:2021 standards BSI (2022), is modeled for each building as follows. Total annual energy consumption [*kWh*] is described as

1 $$\begin{aligned} F_{total} = F_{heating} + F_{DHW} + F_{kitchen} + F_{elec} \end{aligned}$$

where $F_{heating}$ is the total energy consumption from heating the space, $F_{DHW}$ accounts for total hot water usage, $F_{kitchen}$ refers to thermal consumption due to cooking and $F_{elec}$ is the total electricity consumption such as equipment, lighting and any electrically run mechanical systems such as ventilation and cooling. From $F_{total}$ the total operational carbon, $C_o$, [$kgCO_2e$] is estimated for gas, $c_g$ or electricity, $c_e$ as

2 $$\begin{aligned} C_{o}= & c_{g}F_{gas} + c_{e}F_{elec} \end{aligned}$$

$c_e$, may change annually due to the decarbonization of electricity supply. Therefore, $C_{o_{2025-2050}}$ [$kgCO_2e$] is calculated aggregating annual operational carbon values. Detailed quantitative validation of the parametric operational energy modeling against building consumption is available in Abbey et al. (2025), while Sect. 1 of the Supplementary Information SI2 provides validation of key results presented here including total floor area, final energy load and total embodied carbon.

#### Embodied carbon

The embodied carbon of each scenario is calculated based on data from manufactureres and Environmental Product Declarations (EPDs). For each element, all embodied emissions within a A1-C4 boundary as defined by BS EN 15643:2021 standards are calculated (BSI, 2022). The lifespan of each element is used to estimate the number of replacements needed (B4). As a simplification, all end-of-life and in-use emissions from the initial installation are accounted for in the upfront category, and all end-of-life and in-use emissions from any replacements needed are accounted for in the replacement category.

Fabric measures are modeled as increased thermal and ventilation efficiency. These include new insulation systems and replacement of windows. Glass wool and EPS, both among the most common insulation material used in Europe (Pavel et al., 2018), have been modeled due to their relatively low embodied carbon compared to other materials (Grazieschi et al., 2021). The different fabric measures used within this study are aimed to represent typical practice, adopted where appropriate for each building age category. Modeling different insulation systems requires the calculation of thickness [*m*], $d_{ins}$, needed to achieve target U-values [$W/m^2.K$]. $d_{ins}$ allows for the total embodied carbon of each fabric retrofit element, *n*, to be estimated as

3 $$\begin{aligned} C_{fabric,n} = (d_{ins, n}c_{ins} + c_{new, n})A_{n}m_y \end{aligned}$$

where $c_{ins}$ is the embodied carbon factor of insulation [$kgCO_2e/m^3$] and $c_{new}$ is the embodied carbon of any additional elements independent of insulation thickness [$kgCO_2e/m^2$]. $m\in [0,1]$ is the material decarbonization factor, as is a proportion of the current embodied carbon emissions, for the year, *y*, in which retrofit measures are installed–see Sect. 2.2.2.

New mechanical systems require the system size, *Q*, that is replaced. For heating systems, the post-retrofit heat loss coefficient, $U'$ and the maximum temperature difference between outdoor and indoor air is used to estimate the boiler size [*kW*], $Q_{heat}$. The size of the hot water system [*kW*], $Q_{DHW}$, is based on typical occupancy patterns (CIBSE, 2021) so are the size of mechanical ventilation systems [*L*/*s*], $Q_{vent}$ and the maximum required air provision. Therefore, the total embodied carbon of MEP is calculated as

4 $$\begin{aligned} C_{MEP,n} = (c_{n}Q_{n} + c_{new, n}A_{floor})m_y \end{aligned}$$

where $c_{n}$ is the embodied carbon [$kgCO_2e/size$] of each MEP element, *n*, and $c_{new}$ is any new element [$kgCO_2e/m^2$] that is independent of system size. Any replacement emissions [$kgCO_2e$] are calculated as

5 $$\begin{aligned} C_{r}= & N_{r,n}C_{n}m_{(y+l)} \end{aligned}$$

where $N_{r}$ is the number of replacements for each element, $N_{r}=\left\lfloor \frac{l}{s} \right\rfloor$, based on the lifespan [*years*], *l*, and study period [*years*], *s*.

#### Whole life carbon

Figure 1E shows that the whole life carbon [$kgCO_2e$] can be modeled as

6 $$\begin{aligned} C_{wlc}= & \sum _{2025}^{y}C_{ob} + C_{e} + C_{r} + \sum _{y}^{2050}C_{or} \end{aligned}$$

where $C_{ob}$ is the baseline operational carbon emissions [$kgCO_2e$], $C_{or}$ the post-retrofit [$kgCO_2e$] and *y* the year the building is modeled to be renovated.

### Carbon budget calculations

Carbon budgets are used to estimate the required retrofit rate and reduction in greenhouse gas emissions, Fig. 1F. Using the CCC and Tyndall budgets provides a minimum and maximum value to strive for as they have very different interpretations of the total remaining budget for the UK (CCC, 2020; Kuriakose et al., 2022; Li et al., 2022). CCC and Tyndall derived carbon budgets for the English school stock are estimated based on the proportion of emissions currently emitted by this stock type. This methodology is outlined by Li et al. (2022) for English residential buildings and has been adapted for educational buildings, Table 2. One limitation of this method is that both carbon budgets only account for UK emissions, and would not include any emissions from imported materials. As it is currently the case that the majority of materials considered are within UK territorial emissions (Department, 2024; Morrell et al., 2023), we take account of total emissions as though all materials required are manufactured within the territorial UK whether or not imported. We additionally note that carbon budgets are still non-standardized and inherently fuzzy in their scope and sectoral and regional distribution (Kuriakose et al., 2022). The CCC budget, like many others, distribute components of whole life emissions across various sectors with building-specific budgets often only concerning direct and indirect emissions from buildings and those relating to materials typically included in the manufacturing and construction budget. As such, our apportioned CCC budget is pessimistic relative to the CCC’s intent, however, as Anderson et al. (2020) have pointed out, the UK’s planned budget is twice as generous as our fair international contribution to a 1.5-2°C would require. Our consideration of both the CCC and Tyndall budgets allows for a comparison of overall feasibility of retrofit/demolition and the pace required within the inherent uncertainties of the accounting currently possible.

**Table 2:** Estimated 2025–2050 carbon budget for primary/secondary schools and colleges

Budget type	UK	England	Buildings - embodied and operational	All educational stock	Primary/secondary schools and colleges
CCC $[MtCO_2e]$	4533	3677	920	38.0	20.8
Tyndall $[MtCO_2]$	1126	917	229	9.5	5.2

Each column shows how the budget was apportioned based on the estimated proportion of total carbon emissions emitted by the stock type

### New construction benchmarks

New construction benchmarks are used to compare refurbishment with demolition and replacement. This assumes highly energy efficient new construction with all electrified demand. The total predicted whole life carbon of different new construction standards over the 25 year study period is compared to that of the retrofit alternative. This modeling of new construction over the study period uses the same electricity decarbonization scenarios as in Sect. 2.2.2. There are different targets for new construction of educational buildings. From a UK context the key targets have been devised by the Royal Institute of British Architects (RIBA), the London Energy Transformation Initiative (LETI), and more recently UK Net Zero Building Standards, which provide different benchmarks for both embodied carbon and operational energy efficiency to strive for.

One key quality of a building that cannot be easily changed through retrofit is form. Building form measures the size of the external envelope which encloses the internal volume of a space. It can be used to determine the efficiency of a building’s shape and size. The Supplementary Information SI1 explains how different measures of building form are explored in detail for those buildings where demolition may be preferable.

## Results and discussion

### Baseline operational carbon

**Fig. 2:**
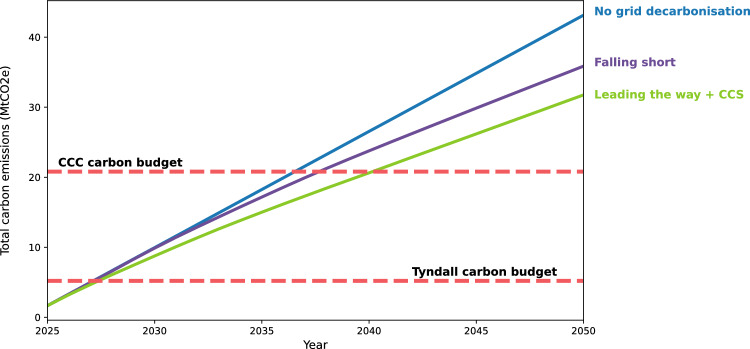
Baseline, pre-retrofit, operational carbon emissions for the English school stock. Estimated 2025–2050 operational carbon emissions for different decarbonization scenarios. Overshoot of the Tyndall budget occurs after 3 years in 2028 with the CCC overshot between 2037–2042 depending on the rate of electricity decarbonization

Figure 2 shows baseline operational carbon emissions for the stock. If we continue using energy at the same rate with no electricity decarbonization, schools would have emitted over 8 times the total Tyndall and 2 times the CCC budget by 2050. Depending on grid decarbonization, emissions are slightly reduced. These values may be higher due to required maintenance of existing buildings which is not included in this study. Even with complete decarbonization of the electricity supply, current reliance on fossil fuels for thermal energy guarantees budget overshoot. Retrofit of existing schools is required to achieve further reductions in emissions.

**Fig. 3:**
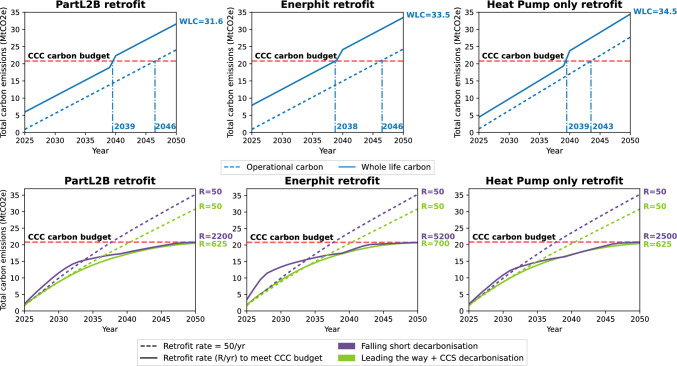
Whole life carbon of the English school stock assuming every building is retrofit in 2025, with no electricity or material decarbonization. The jump in whole life carbon after 15 years occurs due to the modelled replacement of air source heat pumps (Top). The retrofit rates, *R*, to the nearest 25 schools, required to meet the CCC carbon budget compared to current practice (Bottom). The noticeable kinks within the falling short scenario, are due to the upfront embodied carbon cost of annual retrofit and the replacement of heat pumps modelled after 15 years. This is less noticeable for the leading the way model as the retrofit rate is smaller and the upfront carbon cost of MEP is lower

Figure 3 shows cumulative emissions using the no decarbonization scenario. Even if all buildings are retrofit at the start, neither the CCC nor Tyndall carbon budget can be met with operational carbon alone overshooting by $3.2-6.9MtCO_2e$. Results show that successful decarbonization of the stock is only possible with faster decarbonization of the fuel source, here grid electricity. This is in agreement with studies of other countries and building typologies (Berrill et al., 2022; Ghose et al. 2020; Göswein et al., 2021; Hegarty & Kinnane, 2023; Österbring et al., 2019; Verellen & Allacker, 2022).

Comparing the three scenarios shows that the heat pump only model has higher operational carbon emissions caused by a reduction in heat pump efficiency and a lack of fabric improvements. When no decarbonization occurs this difference is significant, emitting over $3MtCO_2e$ more than PartL2B scenario despite lower upfront embodied emissions. While this could be exacerbated by variations in the field performance of heat pumps, our modelled coefficients of performance are on the conservative end compared with recent data on field performance of ASHPs (Energy Systems Catapult, 2023).

### Meeting the CCC carbon budget

The annual retrofit rate required to meet the CCC budget can be calculated using the falling short and leading the way decarbonisation scenarios. The retrofit rate, to the nearest 25 schools, required to the meet the CCC budget is presented in Fig. 3 for each retrofit scenario. Alongside this, a retrofit rate of 50 schools per year is also modelled which represents the UK government’s current goals for school improvements, either through retrofit or rebuild (Department for Education, 2024a). Given the large variation in building ages, condition and funding priorities (Department for Education, 2021) a random selection of schools to be retrofit each year has been modelled.

Figure 3 shows that UK’s planned retrofit rate of 50 schools per year is inadequate for meeting the apportioned CCC carbon budget. By 2050, only 7% of modelled schools will have been refurbished leading to an overshoot of the 2050 budget before 2040. Annual retrofit rates of much higher than 50 schools per year are required to meet the CCC budget for both falling short and leading the way decarbonisation.

The highest required retrofit rate is the Enerphit scenario due to significantly higher embodied emissions. The key difference in embodied carbon is the installation of MVHR and ductwork, $1.8MtCO_2e$, due to large amounts of steel required. This measure also requires additional fan energy consumption compared to naturally ventilated buildings, though the modeled heat recovery does reduce the total heating load. Good ventilation strategies within schools improve cognitive performance (Bakó-Biró et al., 2012), and well-being (Daisey et al., 2003) with mechanical solutions particularly impactful (Hama et al., 2023). If mechanical ventilation is a requirement to maintain good air quality in certain schools, high heat recovery and fabric efficiency are also needed due to the higher embodied cost of these systems.

Figure 3 also shows significantly lower rates for the leading the way scenario, due to lower electricity carbon emissions and differences in refrigerant type. Modeling PartL2B retrofit means, by 2050, 87% of all schools were refurbished. This is in comparison to the falling short model where all stock would be refurbished within 9 years. The fact that not all stock has been retrofit means that in 2050, operational emissions are still above zero despite the negative yearly $c_e$ of $-0.013kgCO_2e/kWh$. This is due to the continued reliance on gas boilers by the stock which has not yet been retrofit and further highlights the need to replace this heating system at scale. The upfront embodied carbon of the heat pump only model is significantly lower than that of PartL2B and Enerphit. This is not the case for replacement carbon emissions due to the increased plant size required to provide heat for less efficient buildings. These higher replacement emissions and increased operational carbon cause the retrofit rate to be higher for the heat pump only scenario when following the falling short decarbonization pathway. When following the leading the way decarbonization pathway, the required retrofit rate is the same for PartL2B and the heat pump only scenario. If the national grid is decarbonized quickly with low embodied carbon heat pumps, it would make no difference which retrofit strategy is used. However, the final energy load is 15% higher for the heat pump only scenario compared to PartL2B, leading to higher annual operational costs. This decarbonization pathway relies heavily on carbon capture and storage. Meanwhile, when modeling future stock scenarios in Sweden to meet governmental targets, the cumulative embodied emissions from new renewable power plants outweighed that of the renovation and new construction of offices and dwellings combined (Francart et al., 2018).

### Meeting the Tyndall budget

**Fig. 4:**
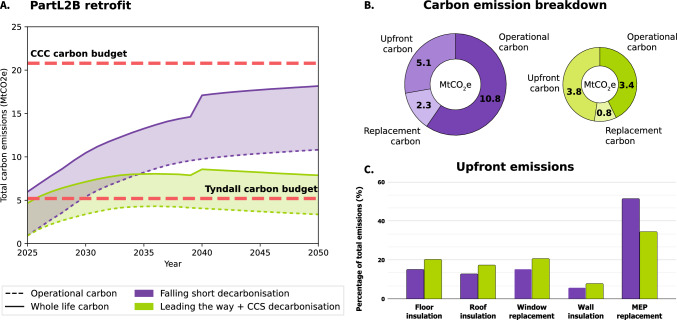
Whole life carbon emissions assuming all English school stock is retrofit to PartL2B standard in 2025 showing total carbon emissions over time, assuming all stock is retrofit in 2025 (**A**), breakdown of total (**B**), and upfront embodied (**C**) carbon emissions for both decarbonization pathways. Note that upfront MEP emissions include refrigerant leakage over the products lifespan

To meet the most stringent carbon budget, the entire stock has been modeled to be retrofit in 2025, as shown in Fig. 4. Only Part L2B retrofit is modeled as this is assumed to be typical, and the previous section shows this retrofit scenario leads to the lowest required retrofit rates.

The falling short decarbonization scenario would not meet the Tyndall budget, even if only operational carbon is modelled. By 2050, whole life carbon emissions are over double the budget which demonstrates the importance of material and electricity decarbonization as well as the urgency with which retrofit needs to be undertaken if we are to limit emissions.

Even using leading the way decarbonization, where by 2037 operational carbon emissions are negative, the Tyndall budget is still not met due to embodied carbon emissions. Operational carbon alone would fall below this budget, but whole life emissions would not. Even if replacement of heat pumps is excluded, this scenario would exceed the budget by $1.9MtCO_2e$ which is over a third of the original budget.

Whole life carbon breakdown shows that material emissions need to be less than $1.4MtCO_2e$ to meet the budget. Optimistically, this is equivalent to less than $20kgCO_2e/m^2$. For context, available retrofit benchmarks assume a light commercial refurbishment without any fabric measures to have an upfront (stages A1-A3) emissions of less than $30kgCO_2e/m^2$ (Bienert et al., 2023). Low carbon materials are then key to meeting the Tyndall budget. This challenge is further demonstrated when considering relevant skills shortage. The Green Jobs Taskforce predict a need for 230,000 more skilled workers by the end of the decade (Green Jobs Taskforce, 2021). The crucial role of local governments and their funding gap has previuosly been discussed (Bevan et al, 2020; LGA, 2024). Considerable increases in funding are needed if carbon targets are to be met.

Occupancy sensitivity analysis is provided in Sect. 3 of Supplementary Information SI2 showing similar patterns between different sensitivity scenarios. The CCC carbon budget cannot be met without multi-sector decarbonization and a marked increase in retrofit rates compared to current practice. The Tyndall budget cannot be met once the impact of upfront and replacement carbon emissions are considered.

#### The significance of MEP embodied carbon

MEP embodied carbon outweighs all other retrofit measures, consistent with observations that MEP can take up 50–75% of projects’ total embodied carbon (Hamot & Bagenal George, 2021). The need for lower carbon heat pumps is clear. The benefits of adopting lower emission refrigerants is also evident with R744 emitting over $1MtCO_2e$ less in upfront embodied carbon compared to R513A. However, this reduction alone is not enough to meet the Tyndall budget and further development of low embodied carbon strategies is necessary, Figure 4. These results also show it would be highly useful to incorporate circular principles within the design of heat pumps as the replacements account for over 10% of the total whole life emissions. Decarbonization of heat pumps’ embodied carbon is especially advisable when considering that heat pumps are central to many governments’ decarbonization strategies (European, 2023; Miller et al., 2023).

#### Material choice

The modeled insulation materials are glass wool and EPS. It is assumed that only the MEP systems require replacement over the study period which is disputable. However, insulation materials do see degradation over time, eg, Stazi et al. (2014) show a 12% degradation in thermal conductivity of glass wool in 25 years, and Acar et al. (2025) show reductions in thermal conductivity of recovered glass wool, stone wool, PUR and XPS. Although, some of this degradation could be due to the end of life recovery process. Rather than imply replacement within the 25 year study period, instead this potential reduction in insulation performance is likely to lead to higher operational energy over time, and potentially further carbon budget overshoot. This highlights a limitation of the study as any potential required replacement of insulation has not been accounted for. Conversely, alternative biogenic insulation materials could be considered to temporarily sequester carbon, making upfront emissions negative if materials are sourced from sustainably managed forests (Hawkins, 2021). These will likely be re-emitted unless further carbon capture or alternative use-cases are facilitated.

### Carbon payback period

**Fig. 5:**
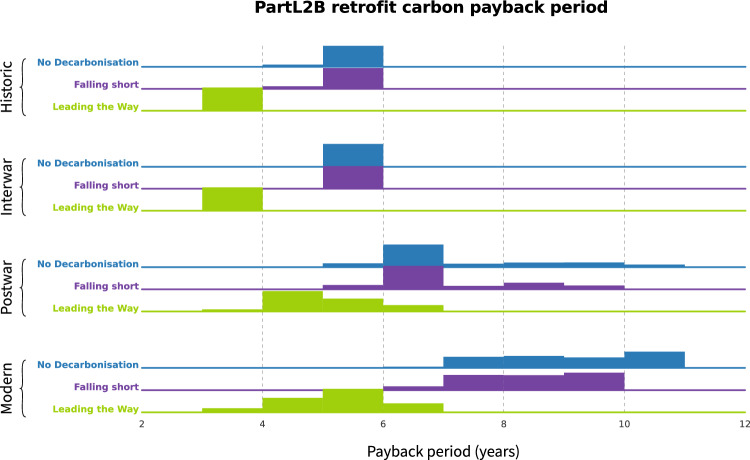
Histograms showing payback periods of buildings for different decarbonization scenarios, assuming PartL2B retrofit in 2025. For each decarbonization scenario the total number of schools within that age category have been modeled. The distributions are relative to the total number of buildings in each age category rather and not the total number of English schools

For each decarbonization scenario, the embodied carbon payback period was calculated, assuming 2025 retrofit to Part L2B standards, Fig. 5. The leading the way scenario has shorter payback periods as this would lead to larger savings between the gas boilers and the heat pumps. Older buildings are shown to have a shorter payback period which demonstrates the benefits of prioritising retrofit of the least thermally efficient buildings. It is clear that all measures are paid back within the 15 year lifespan of heat pumps. This is still the case even in scenarios with no decarbonization. Payback periods are short enough to reduce total carbon emissions for all stock by 2050.

The results show that older buildings, historic and interwar, would need an extended lifespan of more than 7 years and newer buildings, post war and modern, longer than 11 years to justify the operational savings from retrofit. This payback period is significant. Some buildings may have a limited remaining structural lifespan, meaning the building may need structural remediation or demolition before retrofit intervention becomes beneficial. Any required structural remediation within schools may also have significant embodied carbon impacts which would mean that retrofit rates across the stock would need to be increased to still meet the carbon budget.

One limitation of this study is the exclusion of optimal retrofit. This may especially impact modern buildings, which may need less severe interventions to achieve low carbon emissions. For example, external wall and roof insulations require the input of materials that are independent of the insulation thickness. The embodied impact of retrofit is then not merely dependant on the efficiency of the existing building. If a building already achieves a low U-value, it could be beneficial from a whole life carbon perspective to not include these measures as the carbon benefits are reduced.

### Comparison to new construction benchmarks

**Fig. 6:**
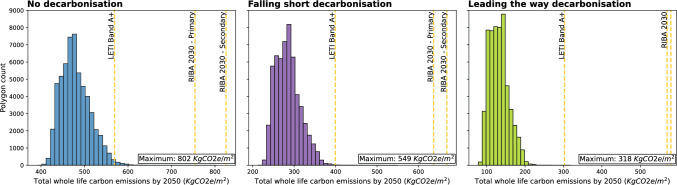
Whole life carbon of retrofit by 2050, assuming they are retrofit in 2025 to PartL2B standards. RIBA and LETI benchmark values for new construction change across each scenario as they are set as energy demand per unit area per year and the modelled decarbonization of electricity impacts the total operational emissions. Results assume that the carbon emissions from the demolition, typically $35kgCO_2e/m^2,$ Sturgis et al. (2023) is already included in the total new construction benchmark

The total internal floor area of school stock is approximately $70km^2$. Multiplying this by the current aspirational embodied carbon target of $540kgCO_2e/m^2$ would be $37.5MtCO_2e$ which is well above the CCC carbon budget for schools. However, it may be the case that for certain building types and forms it would be beneficial from a whole life carbon perspective to demolish them. This is investigated for all buildings within the English school stock with Fig. 6 showing the distribution of total whole life carbon by 2050, of all buildings, assuming they are retrofit in 2025 to PartL2B standards. The importance of considering embodied emissions as well as trying to retain the existing stock is demonstrated as no property has a whole life carbon above that of a new construction with an embodied carbon of $540kgCO_2e/m^2$. This is the level that the Royal Institute of British Architects (RIBA) are striving to achieve today (RIBA, 2021).

Within both the no decarbonization and falling short scenarios, there are a number of properties where whole life carbon falls above the London Energy Transformation Initiative (LETI) A+ target, $260kgCO_2e/m^2$. When exploring the geometric characteristics of these buildings in more detail, a connection between the building form and the total whole life carbon is found. Buildings with a whole life carbon above LETI A+ target have a higher external surface area to internal usable volume. See Sect. 2 in Supplementary Information SI2. The larger external surface area, leads to higher heat losses through the building fabric which limits the improvements from retrofit and leads to a higher yearly operational carbon. It should be noted that we do not account for the different remaining lifespans of new construction and retrofit considering demolition versus refurbishment over a fixed 60 year period (Sturgis et al., 2023).

Over a 60-year period more polygons would have lower overall emissions from replacement to RIBA 2030, $540kgCO_2e/m^2$, standards. However, the majority of floor area would still be retained. When the falling short scenario is modelled only 0.01% of buildings would benefit from demolition. RIBA 2030 is a highly ambitious target and business as usual embodied carbon would be $1000kgCO_2e/m^2$ compared to $540kgCO_2e/m^2$ (RIBA, 2021). Any required structural remediation is not included in this study and therefore poses a limitation to the conclusions formed.

## Conclusion

By modeling whole life carbon emissions of the entire English school stock, retrofit is shown to be pivotal in meeting the devised school’s CCC carbon budget by 2050. Retrofit must occur at markedly increased rates than is currently planned, alongside significant electricity and material decarbonization.

The CCC carbon budget can be met through installation of Part L2B, Enerphit or heat pump retrofit. The more stringent carbon budgets, such as Tyndall, remain very difficult to meet. Cumulative operational carbon can stay within this budget through instantaneous retrofit of all stock and if by 2037, electricity production is actively reducing emissions through carbon capture and storage. However, incorporating whole life carbon, the budget will not be met.

Refrigerant leakage can have significant emissions and the impact from heat pumps is reduced significantly by using a low carbon refrigerant such as R774. Heat pumps still account for the highest proportion of embodied carbon, a hotspot which future research should address. Comparisons to demolition and new construction show that over the 25 year study period, no schools would benefit from demolition from a carbon standpoint, unless an embodied carbon of $260kgCO_2e/m^2$, is achieved. Assuming lifespans of new construction and retrofit match, over a 60 year lifespan retrofit is still preferred for the majority of the total floor area, especially if electricity and material decarbonization is achieved. The carbon payback period of retrofit shows buildings must last longer than 7–11 years to guarantee carbon benefits from retrofit.

## Supplementary Information

Below is the link to the electronic supplementary material.

Supplementary file 1 (PDF 805 KB)

Supplementary file 2 (PDF 737 KB)

## Data Availability

Supporting information which can provide further information for readers is attached to this piece of work as a pdf. All data used for this piece of work is available through the OS Digimaps platform or publicly available datasets as referenced. The supporting code can be found at: https://github.com/ci1hea/whole-life-carbon-stock.
