# Supplementary material for: Actions to decarbonize English schools: a whole life carbon stock assessment
Source: J Ind Ecol. 2026 Feb 26;30(1):391–403. doi: 10.1007/s44498-026-00027-x (PMC13152907; doi:10.1007/s44498-026-00027-x)
Supplement: Supplementary file 1 — (PDF 805 KB) [file 44498_2026_27_MOESM1_ESM.pdf]

Supplementary Information–Methods

Actions to decarbonize English schools: A whole life carbon stock assessment

Danielle Abbey*, Hadi Arbabi*, and Danielle Densley Tingley*†

November 18, 2025

This Supplementary Information provides further data and details for the methodology used within this paper. More specifically, this case study focuses on the whole life carbon impact of retrofit on all English primary, secondary schools and colleges which is approximately 91% of the total school stock, a total of 20,109 buildings. This SI addresses data collection, scenarios modeled and associated whole-life carbon assessment, the estimation of the carbon budgets and benchmarks used for new constructions.

keywords: *industrial ecology; whole life carbon; retrofit; carbon budget; decarbonisation pathways; building material*

*School of Mechanical, Aerospace, & Civil Engineering, University of Sheffield, UK

†d.densleytingley@sheffield.ac.uk

1 Data collection

The main source of data is Verisk's UKBuildings, accessible through OS Digimaps [1]. This service provides a polygon, for each building, containing several key attributes such as building typology, height, area and age.

Figure 1 shows the key methodological stages within the data collection process. As Verisk UK buildings does not distinguish between different educational typologies, the HM Government database was used to provide the Easting/Northing of every school. These coordinates were used to extract relevant schools from Verisk UKBuildings. All educational buildings within the defined search radius of each Easting/Northing were retained with the search radii based on typical outdoor area guidelines for each typology [2]. The Easting/Northing were also used to define the building location and therefore typical monthly weather patterns. Any duplicates were identified with only one polygon retained, designated to the school whose Easting/Northing was closest to the duplicate.

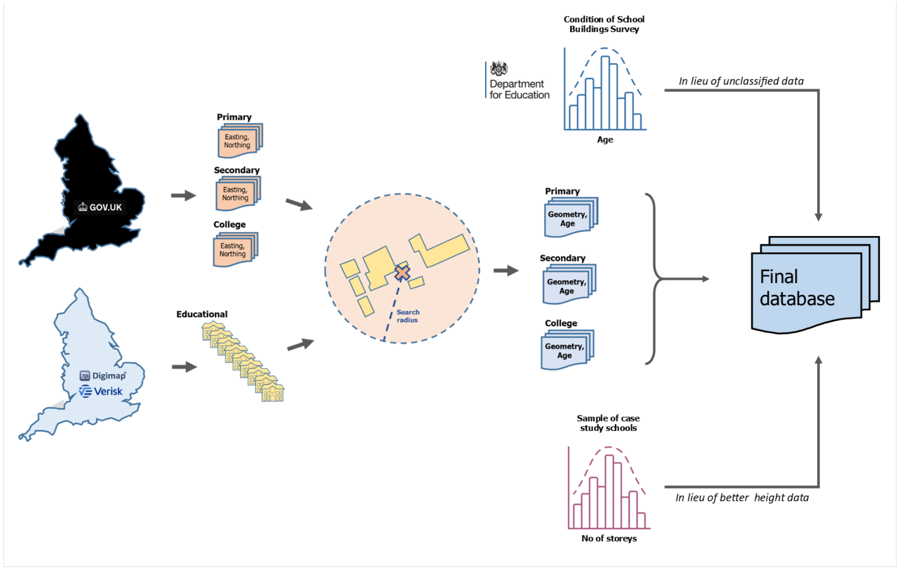

Figure 1: Flow chart to show the data collection process used within this study. Collecting geometry, age and location data for all English primary, secondary schools and colleges.

A distribution of the archetype number of storeys, x, has been applied to the database as this was deemed more accurate than adopting Verisk height data. This decision was made due to analysis of 235 case studies for which x has been collected - see Section 1.1. This does pose a limitation, that though building form is representative at scale, it may not be completely reflective of individual buildings.

The archetype number of storeys, x, alongside Verisk data is used to define key building geometry. Buildings are modelled as a single zone space:

- Plan area $[m^2]$, A_{plan}, is used to define roof area $[m^2]$ and ground floor area $[m^2]$.
- Wall area $[m^2]$, A_{wall} is calculated as:

$$A_{wall} = x * H_{f_f} * P_f \quad (1)$$

  where H_{f_f} is the total floor to floor height $[m]$ and P_f the exposed perimeter $[m]$. This in turn can be disaggregated into wall and window area using the glazing ratio, g.

- Internal floor area $[m^2]$ is defined as:

$$A_{floor} = x * A_{plan} * G_r \quad (2)$$

  where G_r is the typical gross internal ratio, which is used to account for walls taking up a proportion the floor area.

- Internal volume is defined as:

$$V = N_{floors} * H_{f_c} * A_{plan} * G_r \quad (3)$$

  where H_{f_c} is the floor to ceiling height.

1.1 Defining building height

Geometry is a very important factor within whole life carbon modelling as it defines not only the total heated floor area but the total surface area and volume in which heat is lost from the building. It also impacts total embodied carbon as it defines the amount of material that needs to be added.

235 school case studies were assessed to understand the quality of Verisk UK buildings data.

Figure 2 provides evidence that Verisk height data is not accurate. Over 25% of polygons within the database had a Verisk height of 17.8m while actually being 1 storey tall. Also, over 10% of polygons had a height less than 2m despite typical floor to ceiling heights ranging from 3m upward [3]. This implies significant issues with data quality for building height.

17.8m is found to be the Verisk archetype height, used when other data is unavailable. However, especially for primary schools who typically have only one storey [4] this number is not acceptable and would lead to a large overestimation in energy consumption and floor area.

For these reasons, an archetype height, x, will be used - applied as a distribution to the English schools stock. In lieu of better data the distribution of x is based on the results of the 235 case studies. Using this has also been assessed against the total predicted floor area of the school stock [5] and was found to lead to results within 5% of this value.

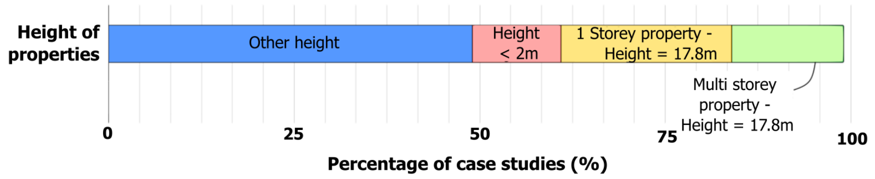

Figure 2: Bar chart to show potential issues with Verisk height data for the 235 case study schools.

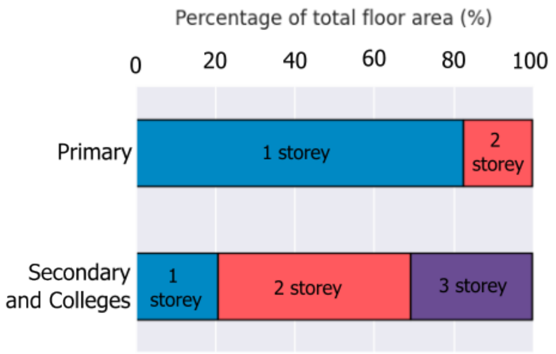

Figure 3: Bar chart to show the distribution of number of storeys within the 235 case study schools.

1.2 Baseline building performance

Building age will be used to define the baseline performance of each building. Table 1 shows the incremental improvements to English building regulations within the last 60 years. Infiltration rates are split pre and post 2002 as this was when airtightness regulations were first introduced [6]. Data for buildings built pre-building regulation was found from existing literature [7, 3].

Verisk UK buildings provides age categories as shown in Table 1, and shows that each category can span several building performance levels. A significant proportion of data points were also found to be unclassified in age.

Therefore, as shown in Figure 1, a distribution will be applied across any unclassified, post war or modern data based on the proportion of actual ages [5]. The school condition survey provides a more detailed breakdown of ages as a percentage of the total school stock [5].

Age		U-value $[W/m^2K]$					
Verisk	Building performance category	Wall	Window*	Floor	Roof*	H_{f_c} $[m]$	Infiltration Rate $[hr^{-1}]$
Historic	Pre 1919	1.7	2.2	1	1.1	4.5	0.45 - 0.675 †
Interwar	1919-1945	1.5	2.2	1	1.1	3.5	
Post war	1945-1976	1.7	2.2	1	1.4	3	
	1976-1985	1	2.2	0.94	0.55		
	1985-1990	0.6	2.2	0.6	0.35		
	1990-2002	0.45	2.2	0.45	0.25		
Modern	2002-2010	0.4	2.2	0.25	0.25	3	0.25 - 0.35 †
	2010-2021	0.35	2.2	0.25	0.25		
	2021-2023	0.26	1.6	0.18	0.18		
References: [6, 8, 7, 9, 3, 1]							
† Value depends on number of floors<br>*A minimum performance is assumed due to the lifespan of these elements.							

Table 1: Inputs for building performance of baseline, pre retrofit, schools. Different age categorisations within the model are used to define building performance.

All existing buildings are assumed to run on gas boilers as is typical for this stock type [10]. The efficiency of this boiler is assumed to be 0.8, which is higher than would be typical of very old boilers [11]. As the lifespan of a boiler is usually 15-20 years, all existing boilers are assumed to have a minimum level of efficiency [12]. This principle has also been assumed for the U-value of windows and flat roof systems as the lifespan of both these elements is typically 30 years [13, 14]. Pitched roof modelling is also assumed to have some form of insulation, even pre-building regulation, as loft insulation is an already commonly installed retrofit measure [15].

The glazing ratio for each building is assumed to be a constant value of 26% which is typical for schools [16].

1.3 Retrofit scenarios

Three typical retrofit scenarios have been modelled to understand the impact of different refurbishment measures. These are titled **PartL2B, Enerphit and heat pump only**, with key performance data demonstrated in Table 2.

Input	Part L2B	Enerphit	Heat Pump only	References
Wall U-value $[W/m^2K]$	0.35/0.3	0.35/0.15	-	[9, 17]
Glazing U-value $[W/m^2K]$	1.6	0.6	-	[9, 17]
Roof U-value $[W/m^2K]$	0.16	0.15	-	[9, 17]
Floor U-value $[W/m^2K]$	0.25	0.15	-	[9, 17]
Infiltration rate $[hr^-1]$	0.3	0.05	-	[18]
Heat recovery efficiency	-	0.9	-	[19]
Heating efficiency	3.4	3.4	2.3	[20, 21]
Hot water efficiency	2.3	2.3	2.3	[20, 21]

Table 2: Key building performance metrics for each retrofit scenario. U-values vary within wall category to account for internal or external insulation systems [17].

Part L2B follows UK building regulation standards for non-residential retrofit, with the key requirement being improvements to the building fabric. Enerphit, the retrofit equivalent to Passivhaus, requires strict levels of airtightness and fabric efficiency. For this reason mechanical ventilation and heat recovery (MVHR) is advised to ensure safe levels of ventilation [17].

As neither Part L2B nor Enerphit standards define a specific heating or hot water system, this is assumed to be a replacement of all fossil fuel systems with a heat pump. Medium temperature heat pumps are modelled that can replace these systems with no change to emitter size, due to the improvements to fabric efficiency [22]. In contrast to this, the heat pump only scenario has no fabric efficiency improvements and therefore assumes a high temperature heat pump.

1.4 Decarbonisation scenarios

Three different decarbonisation scenarios have been developed, titled no decarbonisation, falling short, and leading the way. These scenarios model different levels of electricity and material decarbonisation.

Electricity decarbonisation is modelled using the UK national grids Future Energy scenarios [23] which predict different levels of decarbonisation for electricity by 2050. The chosen scenarios have been demonstrated in Figure 4,

which provide worst, best and typical values of c_e $[KgCo2e/Kwh]$. For gas, a constant value of c_g of $0.18KgCo2e/kWh$ is used [24].

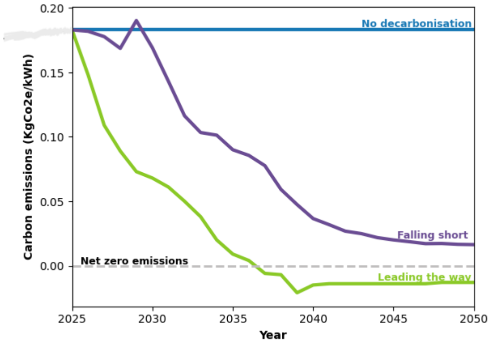

Figure 4: Different electricity carbon factors used for operational carbon modelling. **No decarbonisation** models constant emissions from electricity. **Falling short** follows the UK national grids falling short model, excluding the impacts of carbon capture and storage. **Leading the way** is the most optimistic model provided by the UK national grid and includes carbon capture and storage [23].

Alongside this, simplified **material decarbonisation** will also be modelled. Material decarbonisation is hard to quantify as it relies on many different factors, including electricity decarbonisation and the implementation of material specific, low carbon strategies. Decarbonisation measures differ widely between different materials [25] and there are many different materials used in construction.

For this piece of work, the decarbonisation pathways for 3 key materials within retrofit installations are accounted for. These materials are steel, glass and insulation. Decarbonisation data is gathered from the government's industrial decarbonisation pathways [25], which outline different levels of success in decarbonising these 3 materials. Each pathway has been matched to the scenarios outlined for electricity decarbonisation, as described in Figure 5.

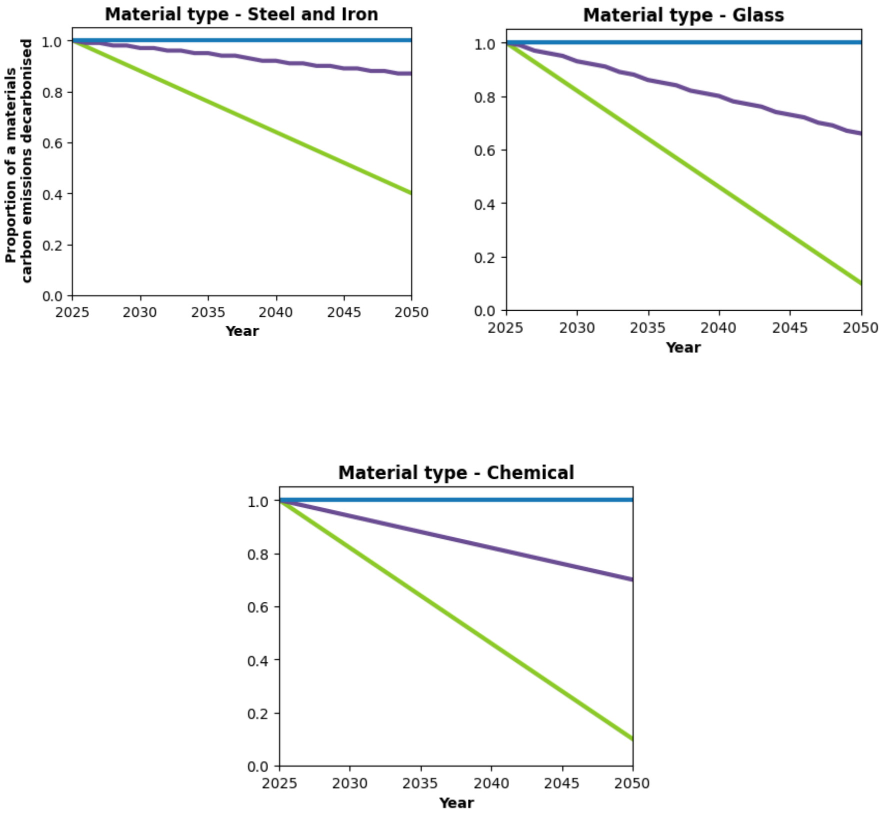

Figure 5: Graphs to show how material decarbonisation was modelled for key retrofit materials [25]. Graphs represent embodied carbon of each material per year as a proportion of current emissions. Blue highlights a **no decarbonisation** model, purple a business as usual scenario (equivalent to **falling short**) and green the max technical scenario including carbon capture (equivalent to **leading the way**).

Each retrofit element will follow the decarbonisation pathway for the material which takes up the majority of that element's total mass. For example, EPDs show that Steel and Iron take up 44 - 78% of heat pump's and 66 - 80% of MVHR's total mass [A sample of all available commercial data from the PEP database [26]. Air to water heat pumps from brands Aldes, Ariston, CIAT, Panasonic, and Mitsubishi. MVHR systems from brands Atlantic and Vim.]. Therefore, MEP decarbonisation, with exception of refrigerant choice, will be based on the predicted decarbonisation pathway of Steel and Iron [25] in Fig-

ure 5a. This is a clear simplification, showing why different sensitivities of this assumption are required. These different sensitivities are provided in Figure 5.

Another scenario that will be included is refrigerant choice. Both the no decarbonisation and falling short scenarios will be assumed to use typical refrigerant of R513A. However, to understand the impact of low carbon materials, the leading the way scenario will be modelled using R774 refrigerant which has significantly lower emissions.

1.5 Occupancy sensitivity analysis

Another potential cause of model error are differences in occupancy behaviour to what has been modelled in this body of work. For example, those buildings with lower heating loads may be able to afford higher set point temperatures and usage patterns. When this occurs post retrofit it is known as the rebound effect [27].

Also, reductions in energy consumption due to occupancy behaviour changes have been shown to be beneficial to meeting government targets with a Norwegian study showing reductions in domestic hot water consumption are important for meeting a 50% reduction target [28].

With these two things in mind, it is important to conduct sensitivity analysis on potential changes to occupancy. When it comes to a potential performance gap, for modelling purposes we have assumed that post retrofit performance matches what is modelled.

The chosen occupancy sensitivity scenarios will be with further detail in Table 3:

- **Minimum:** $17°C$ average set point temperature with a heating season from November to the end of March.
- **Typical:** $19°C$ average set point with a heating season from mid October to mid April.
- **Maximum:** $21°C$ average set point with a heating season from the beginning of October to end of April.

Results are provided in SI. of the Supplementary Information–Results.

2 Whole life carbon modelling

2.1 Operational carbon

Energy consumption is modelled for each building as follows. Total yearly energy consumption $[kWh]$ is described by splitting it into its component parts:

$$F_{total} = F_{heating} + F_{DHW} + F_{kitchen} + F_{elec} \tag{4}$$

**Value**	Typical	Range	Notes
Set point temperature ($°C$)	19	$\pm 2°C$	CIBSE Guide A provides a range of 19 - 21$°C$ [8] while the NEU stipulates a minimum temperature of 16$°C$ for a school and 18$°C$ for a classroom [29].
Heating season ($month$)	Mid Apr. - Mid Oct	±1 mon.	Literature shows the heating season is likely to vary [30], with data claiming half a month different on each side of the heating season (**1 month total**).
Hot water usage (L/day)	0.73/1.99	±15%	Domestic hot water usage is shown to vary typically by 15% which has been used in lieu of more specific data [31].
Kitchen gas usage ($kWh/meal$)	0.41	±33%	CIBSE TM50:2021 [32] claims kitchen use varies between 6-12kWh/m^2 so we assume a 33% variation.

Table 3: Different modelled changes to occupancy behaviour during sensitivity analysis.

where $F_{heating}$ is the total energy consumption from heating the space, F_{DHW} accounts for total hot water usage, $F_{kitchen}$ refers to thermal consumption due to cooking and F_{elec} is the total electricity consumption such as equipment, lighting and any electrically run mechanical systems such as ventilation and cooling.

The vast majority of the modelled stock type is naturally ventilated [10], and this study assumes that this is the case for all baseline buildings. Therefore, total electricity consumption, F_{elec}, in this study describes only occupant related impacts such as lighting and equipment usage. However, the whole life impact of mechanical ventilation will be explored as a retrofit measure.

To estimate heating energy consumption, the degree days model will be used, which is shown to be a computationally efficient model, ideal for early stage design decisions where knowledge of input data is limited [33].

This model relies on the calculation of a base temperature [C], θ_b, which is defined as the external temperature where no mechanical heating is required due to the balance between heat being lost, through fabric and ventilation, and gained, through internal and solar gains. Subtracting the average outdoor temperature from this value represents the total temperature difference in which mechanical heat is required to provide a comfortable space. Therefore, the total fuel consumption to be estimated as:

$$F_{heating} = \sum_{m} \frac{24N_{days}U'}{\eta} \frac{(\theta_b - \theta_{om})}{1 - e^{\frac{2.5}{\sigma_\theta}(\theta_b - \theta_{om})}} \quad (5)$$

where N_m is the number of days in each month of the heating season, η is the efficiency of the heating system, $\theta_{o,m}$ is the monthly average outdoor temperature [$^\circ C$]. This method accounts for temperature fluctuations on a monthly basis [33] which has its advantages over extremely simplified methods. The denominator, $1 - e^{\frac{2.5}{\sigma_\theta}(\theta_b - \theta_{om})}$, is a correction factor proposed by Hitchin [33] to estimate the impact of varying temperatures throughout the month.

The total heat loss coefficient [W/K], U', can be defined as:

$$U' = \frac{\sum_n A_n U_n + \frac{1}{3}NV}{1000} \quad (6)$$

Where U is the U-value [W/m^2K] of each element, n, and therefore used to describe heat lost through the building fabric and N is the total ventilation and infiltration losses [hr^{-1}] so used to describe the exchange of internal with external air. Calculations of θ_b follow the methodology outlined in CIBSE Guide TM41:2006, assuming the building is intermittently occupied [33].

Hot water (DHW) usage is estimated through [34, 35]:

$$F_{DHW}(kWh) = \sum_{m=1}^{12} \frac{4.18}{3600\eta_{DHW}} \Delta\theta_m * N_{days} * L * A_{floor} \quad (7)$$

Where η_{DHW} is the total efficiency of the hot water system, including distribution and system losses and boiler efficiency. ΔT_m [K], is the temperature difference between incoming cold and outgoing hot water, which varies monthly [35]. L [L/m^2] is an area weighted average of total typical hot water production for all different types of zones within a school. The kitchen energy benchmark outlined below includes hot water consumption for kitchen related activities and, therefore, this zone is not accounted for in F_{DHW}.

Kitchen thermal energy consumption, $F_{kitchen}$, includes all the processes required for cooking - such as gas stove usage and hot water consumption. This is calculated using a typical benchmark provided by CIBSE TM50:2021, G, [$kWh/meal$] [32]:

$$F_{kitchen} = N_{meals}\Phi_{kitchen} \quad (8)$$

The number of meals, N_{meals}, was calculated as:

$$N_{meals} = A_{floor} * o * N_{days} * M_r \quad (9)$$

Where N_d, is the total occupied days [$days$], o the occupancy density [$people/m^2$] and M_r which is the proportion of people within the building that want a meal.

This study focuses on thermal energy retrofit and therefore electricity consumption, $F_{electricity}$, does not need to be split between lighting and equipment usage. $F_{electricity}$ needs to be in the correct magnitude to assess whole life carbon emissions fairly.

Display Energy Certificates (DECs) provide metered energy consumption for all public building's above $1000m^2$ which is also split up based on the CIBSE TM46 typologies [36]. The entirety of England and Wales 'Schools and seasonal public buildings' within the DECs database was downloaded. Repeated DECs were removed using the postcode, with only the most recent DEC for each postcode retained. Also, only 'heated and naturally ventilated buildings' whose heating did not run off electricity were isolated so that only small power, lighting and unregulated consumption was included in the electricity data. This left 48272 schools and seasonal public buildings.

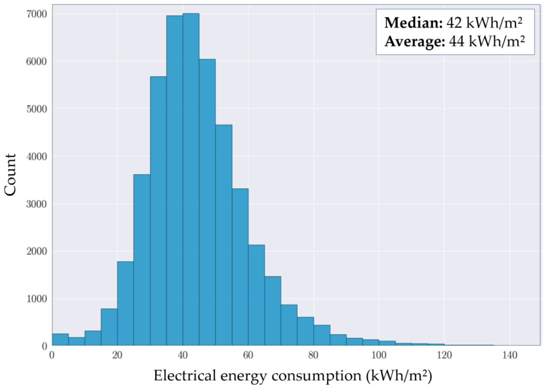

Figure 6: Histogram to show the distribution of energy consumption, within the naturally ventilated schools and seasonal public buildings in the DECs database.

The median and average values shows that schools have a typical electricity usage very similar electricity consumption to that of the CIBSE TM46:2008 benchmarks [37], $\Phi_{elec} = 40$, which allows for electricity consumption to be estimated:

$$F_{elec} = \Phi_{elec} * A_{floor} \tag{10}$$

For any retrofit scenarios where a ventilation system is installed, additional electricity consumption will be estimated using the Specific Fan Power of the system $[kW/m^3s^{-1}]$ multiplied by the estimated total hours of usage each year and typical air flow $[m^3/sm^2]$ [38]. From yearly energy consumption estimations, total operational carbon $[KgCO2e]$ can be estimated through multiplication of the respective carbon factor $[KgCO2e/kWh]$, of either gas, c_g or electricity, c_e:

$$C_o \quad = \quad c_g F_{gas} + c_e F_{electricity} \tag{11}$$

c_e, may change each year due to the decarbonisation of electricity supply. Total C_o $[KgCo2e]$ is calculated by summing yearly operational carbon over the appropriate study period, which in this case is 25 years from 2025 - 2050.

2.2 Embodied carbon

The embodied carbon of each retrofit scenario is now calculated by collecting appropriate data from Environmental Product Declarations (EPDs), manufacturer's data and scientific journal papers. For each element this was estimated from an A1 - C4 boundary as defined by BS EN 15643:2021 standards [39]. The lifespan of each building element was used to estimate the number of replacements which occur over the 25 year period (B4).

Embodied carbon will be split into upfront and replacement emissions (B4). As a whole life carbon assessment is undertaken, as a simplification, all end of life and in-use emissions are included within either the upfront or replacement emission category.

Fabric measures require the calculation of the insulation thickness needed to achieve the new design U-value $[W/m^2.K]$, U_{new}. Glass wool and EPS have been chosen as the main insulation materials due to their relatively low embodied carbon [40] compared to other materials. The required thermal resistance $[m^2.K/W]$, R_{ins}, of each new element can be calculated as:

$$R_{ins} = \frac{1}{U_{new}} - R_{ex} - R_{in} - R_{old} - R_{new} \tag{12}$$

where R_{ex} and R_{in} are the external and internal resistances of the element [8], R_{old} is the thermal resistance of the existing element and R_{new} is the thermal resistance of any new elements that are independent of insulation thickness. Examples of R_{new} include render for external wall insulation and the waterproof membrane used for flat roof insulation.

The thickness of the insulation can now be calculated as:

$$d_{ins} = R_{ins}\lambda \tag{13}$$

using the thermal conductivity $[W/m.K]$, λ, of the insulation. This allows for the total embodied carbon of each fabric retrofit element, n, to be estimated as:

$$C_{fabric,n} = [d_{ins}c_{ins} + c_{new}]A_n m_y \tag{14}$$

where c_ins is the embodied carbon factor of insulation $[KgCo2e/m^3]$ and c_{new} is the embodied carbon of any additional elements independent of insulation thickness $[KgCO2e/m^2]$. m is the material decarbonisation factor for the year, y in which retrofit measures were installed. This value is between 0 and 1 as is a proportion of the current embodied carbon emissions.

Table 4 shows the different fabric measures used within this study, aimed to represent typical practice, adopted where appropriate for each building. External wall insulation is only used for buildings built post 1945. The typology of each roof is also based on building age [7]. All buildings are assumed to be solid floor [6] and the floor insulated through the addition of insulation and screed onto the existing slab.

Retrofit measure	Material(s)	Embodied carbon	Unit $[kgCO_2e/unit]$	Lifespan $[years]$	
Loft insulation	Glass wool - roll	10.9	m^3	> 50	[41]
Flat roof insulation	EPS insulation	67.1	m^3	> 50	[42]
	Roof waterproof covering	6.6	m^2	30	[43]
Floor insulation	EPS insulation	67.1	m^3	> 50	[42]
	Screed and VCL	20.4	m^2	> 50	[44, 45]
External wall insulation	EPS insulation	67.1	m^3	> 50	[42]
	ETICs components	4.8	m^2	> 50	[45, 46, 47]
Internal wall insulation	Glass wool - slab	19.8	m^3	> 50	[48]
	Timber frame	4.6	m^2	> 50	[45, 46, 47]
Double glazing	PVC frame	58.3	m^2	30	[49]
Triple glazing	PVC frame	78.2	m^2	30	[50]

Table 4: Embodied carbon for different fabric retrofit measures (A1 - C4 boundaries excluding stages B6, B7 and B4). Lifespan of each element is provided to understand the typical number of replacements (B4) that would occur over the study period or building lifespan.

New **Mechanical systems** require the calculation of the system size, Q, that needs to be replaced. For heating systems, this is undertaken using the post retrofit heat loss coefficient, U', the maximum temperature difference between outdoor and indoor air and a conservative overestimation factor, β, which is typical practise when designing new systems [33, 8]:

$$Q_{Heat} = U'(\theta_{sp} - \theta_{winter})\beta \tag{15}$$

The size of the hot water system, Q_{DHW}, is estimated by calculating the size

required to provide sufficient hot water to the occupant based on occupancy patterns [8].

Retrofit measure	Material(s)	Embodied carbon	Unit $[kgCO_2e/unit]$	Lifespan $(years)$	
Air Source Heat pump	Type: R513A	188	KW	15	[51]
Air Source Heat pump	Type: R774	90	KW	15	[52]
Heating distribution	Heating systems only	0.7	m^2	45	[53]
Low temperature emitter	Steel	155*	KW	60	[54, 55]
MVHR	MVHR	3.74	$L.s^{-1}$	15	[52]
	Ductwork	14.8	m^2	40	[56, 57]

Table 5: Embodied carbon (A1 - C4 boundaries excluding stages B6, B7 and B4) data for MEP measures modelled. Lifespan of each element is provided to understand the typical number of replacements (B4) that would occur over the study period. *Calculated by finding the embodied carbon per m^2 of emitter ($kgCO_2e/m^2$) and the total heat output at low temperature ($875W/m^2$) [58].

Ventilation system embodied carbon data is provided in $kgCO2e$ per Ls^{-1}. Therefore a predicted maximum size for the system $[Ls^{-1}]$ was calculated as:

$$Q_{vent} = A_{floor}ov \tag{16}$$

where o is the maximum occupancy density of the space $[people/m^2]$ and v the typical air provision per person $[L/s.person]$. From these values the total embodied carbon of MEP can be calculated as:

$$C_{MEP,n} = (c_n Q_n + c_{new} A_{floor}) m_y \tag{17}$$

where c_n is the embodied carbon $[KgCO2e/size]$ of each MEP element, n, and c_{new} is any new element $[KgCO2e/m^2]$ that is independent of system size. Examples include the new distribution system for the heating, hot water and mechanical ventilation.

The embodied carbon of each retrofit element can then be summed to estimate the total upfront embodied carbon $[KgCo2e]$, C_e.

Any replacement emissions $[KgCo2e]$ can also be calculated as:

$$C_r = N_r C_n m_{(y+l)} \tag{18}$$

where N_r is the number of replacements for each element, $N_n = \lfloor \frac{l}{s} \rfloor$, based on the lifespan [$years$], l, and study period [$years$], s.

2.3 Whole life carbon

The whole life carbon [$KgCo2e$] can then be modelled as:

$$C_{wlc} = \sum_{2025}^{y} C_{ob} + C_e + C_r + \sum_{y}^{2050} C_{or} \quad (19)$$

where C_{ob} is the baseline operational carbon emissions [$KgCo2e$], C_{or} the post retrofit [$KgCo2e$] and y the year the building is modelled to be retrofit.

3 Modelled scenarios

Occupancy data is important to understand key consumption values such as internal temperature, total internal gains to the space, typical hot water, and kitchen gas usage. Inputs related to occupancy and usage are split between primary, secondary schools and colleges.

The chosen values reflect a typical occupancy pattern for each typology, using CIBSE Guide A [8] and guidelines for schools [59].

Weather data is used to define monthly outdoor temperatures [60] and solar gains to the space [61] for different regions of England. A comprehensive outline of all the data used within this study is provided in Appendix A below.

It is acknowledged that occupancy typically differs between schools. There is also evidence of changes to occupancy behaviour post retrofit. Due to a higher building thermal efficiency, energy usage can be increased by the occupant without additional costs [27]. This is known as the rebound effect. Therefore, sensitivity analysis for potential differences in occupancy have been provided in Section 1.5.

4 Carbon budget

To estimate the carbon budget for the English school stock, the methodology outlined by Li et al., [62] has been adapted for educational buildings. Two estimates for the carbon budget have been calculated using the CCC [63] and the Tyndall reports [64].

The CCC carbon budget is the estimated required reduction in greenhouse gases, consistent with the Paris Agreement, which the UK Government has also set its carbon budget in line with [63].

The Tyndall budget presents recommended climate change commitments for UK local authorities that are also aligned with the commitments in the Paris Agreement [64].

These two budgets for the UK [$MtCO2e$], B, provide a minimum and maximum value to strive for.

Carbon budget data, as in Table 6, is aggregated to calculate the total carbon budget for the UK as:

$$B_{2025-2050} = \frac{3}{5}C_{2023-2027} + B_{2028-2032} + B_{2033-2037} + B_{2038-2042} + B_{2043-2047} + \frac{3}{53}B_{2048-2100} \quad (20)$$

	**CCC - UK**	**Tyndall - UK**	**Tyndall - England**	**Proportion - $\frac{England}{UK}$ (%)**	**CCC - England**
2023 - 2027	1950	742	601	80.8	1575
2028 - 2032	1725	367	298	81.0	1398
2033 - 2037	965	181	148	81.3	785
2038 - 2042	491*	89.6	73.2	81.6	400
2043 - 2047	182*	44.3	36.3	81.7	148
2048 - 2100	15.5*	43.3	35.7	82.2	12.7
2025 - 2050	4533		917	-	3677
	*This data was interpolated directly from Figure .. of the CCC 6th carbon budget using the 'Balanced Net Zero Pathway'. These values assume linear reduction of yearly total carbon emissions within each period [63].				

Table 6: Carbon budget calculations from 2025 - 2050, using the CCC 6th carbon budget calculations [63] and Tyndall budgets [64].

Calculations to apportion the carbon budget use estimations of the total carbon emissions currently emitted by English primary, secondary schools and colleges:

$$B_{Schools} = B_{UK} * f_{England} * f_{Buildings} * f_{Education} * f_{Schools} \quad (21)$$

where $f_{England}$ is the proportion of UK emissions attributed to England based on the Tyndall budget, as shown in Table 6 [64], $f_{buildings}$ is the proportion of emissions attributed to buildings (including both operational and embodied carbon) [65], $f_{Education}$ is the proportion of total building emissions attributed to all public educational buildings, including Universities and schools [66, 67]. Finally, $f_{Schools}$ is the proportion educational emissions attributed to primary, secondary schools and colleges. $f_{Schools}$ has been calculated using the proportion of total floor area weighted by typical carbon emissions - see Section 4.1.

**Carbon budget**: 2025 - 2050					
**Budget type**	UK	England	Buildings - embodied and operational	All educational stock	**Primary, secondary schools and colleges**
**CCC** [$MtCo2e$]	4533	3677	920	38	**20.8**
**Tyndall** [$MtCo2$]	1126	917	229	9.5	**5.2**
References:	[63, 64]	[64]	[65]	[66, 67]	

Table 7: Table to show estimated total carbon budget from 2025 - 2050 for primary, secondary schools and colleges. Two estimates for the carbon budget have been included based on different reports - the CCC and Tyndall.

One limitation of this method is that the CCC and Tyndall budget only account for UK emissions, and therefore would not include any imported material emissions. However, data from the Department for Levelling Up, Housing & Communities [68] shows that the majority of building materials would likely be accounted for using these methods [69]. Also, there are specific sectors in the UK that may be harder to decarbonise than others, meaning it would be fair to apportion higher levels of the carbon budget to these sectors than they currently contribute to.

4.1 Estimating $f_{Schools}$

In this study, the impacts from university buildings and nurseries are excluded. Only the impact of primary, secondary schools and colleges must be accounted for. Therefore Table 8 shows how a proportion of the total educational stock has been developed based on the total floor area of each space weighted by the typical benchmark carbon emissions [37]. **This value is 54.7%**.

**School Type**	**Floor Area** (m^2)	**Carbon benchmark** ($KgCo2e/m^2$)	**Proportion (%)** (Carbon and area weighted)
Primary	33,272,454	50.5	54.7
Secondary	37,157,716	50.5	
Colleges	1,559,110	50.5	
Special	3,766,418	50.5	45.3
All-through	2,054,922	50.5	
PRU	334,130	50.5	
Nursery	300,030	50.5	
Alternative provision	221,457	50.5	
University stock*	29,866,350	89.6	

Table 8: Calculation procedure for the carbon and floor area weighted proportion of primary, secondary schools and colleges [37, 5]. *Area estimated - 141/168 universities including the 50 most populated universities in the country [70, 71, 72, 73, 74, 75].

5 New construction comparison

New construction benchmarks will be used in this study to compare refurbishment to the alternative option of demolition and replacement. This will assume a highly energy efficient new construction where all energy is provided through electricity.

There is different benchmark data and targets for new construction of educational buildings, demonstrated in Table 9. Comparing these benchmarks shows there is a large spread in results, especially within embodied carbon targets. For context, the RIBA 2030 target is something that the organisation claim the industry should be striving to achieve today, but current Business as Usual values imply this is not regularly achieved [76].

To estimate the whole life carbon of new construction over the 25 year study period, the same electricity decarbonisation scenarios will be used to model operational carbon. As data in Table 9 has a system boundary of A1 - C4 [77, 19, 76], any potential impacts of maintenance, replacement and material decarbonisation are assumed to be included within each benchmark.

Source	Target type	Embodied carbon A1-C4 [$KgCo2e/m^2$]	Operational energy [$Kwh/m^2.yr$]
RIBA 2030 climate challenge [76]	Business as Usual	1000	130
	2020	675	70 - secondary<br>55 - primary
	2030	540	60 - secondary<br>45 - primary
LETI carbon targets [77, 19]	Band C - 2020	675	65
	Band A + Not yet a target	260	65

Table 9: Different new construction embodied carbon and operational energy benchmarks, which define different levels of construction buildings could strive to achieve.

5.1 Understanding building form

In this study, the key characteristics of any buildings which would benefit from demolition are to be understood.

One key quality of a building that cannot be changed easily through retrofit is building form. Form aims to measure efficiency in which the external envelope encloses the internal volume of a space. Therefore measures of building form can be used to understand the level of efficiency of a building's shape and size. Two dimensionless measures of form which have been used in past work [78] are slenderness, k, and aspect ratio, r, which are defined as:

$$k = H/L \tag{22}$$

$$r = W/L \tag{23}$$

where W is the width $[m]$, L the length $[m]$ and H the height $[m]$ assuming rectangular form.

Both these factors can be used to define key characteristics of the building and the efficiency of its form. For example, for buildings of the same internal volume, as k increases so does the external surface area, leading to higher heat losses. r helps define the efficiency of the building footprint. For buildings of the same plan area, as r differs from a square plan, where $r = 1$, the amount of exposed surface area increases.

These values have been chosen to be investigated because different building elements have different fabric efficiencies. Therefore, by keeping these values separate we can more easily understand the specific impact of different wall, floor and roof areas in existing buildings.

The distribution of k and r for entire stock will be compared to those buildings where demolition and replacement may be preferable. This should show what key characteristics these buildings have, and what impact an inefficient form can have on this comparison.

Calculation of these factors, requires the assumption of rectangular form to estimate Width, W, and Length, L, and Height, H, as follows:

Height $[m]$ can be taken directly from existing geometry data, based on an archetype number of storeys, x.

Length $[m]$ must be estimated for each building, assuming that it is of rectangular form. This was completed using the minimum rotated rectangle function, which returns the rectangular shape of the minimum area which completely bounds the polygon as shown in Figure 7. The Length is then estimated as the maximum value out of the four sided shape.

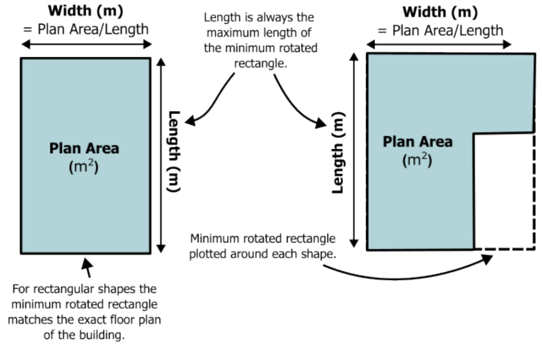

Figure 7: A sketch to explain how Length and Width were calculated for all shapes.

Width $[m]$ can now be estimated by dividing the plan area by the length. This ensures that building floor area is still accurate (See Figure 7).

$$W = A_{plan}/L \tag{24}$$

6 Energy modelling data

Inputs for energy modelling used in this study are now outlined (See Table 10).

The setpoint temperature is set as $19°C$. This figure was chosen as the minimum internal temperature in schools aimed at $18°C$. It is not expected that perfect occupancy occurs at all times, so choosing a value slightly higher than this is deemed sensible. Typical internal gains and hot water usage were calculated using an area weighted average of a typical space breakdown for a school [59]. Internal gains were estimated from CIBSE Guide A [8]. Kitchen water usage is included in within the typical kitchen gas usage (κ) [32]. Therefore any kitchen hot water usage is excluded from the calculations.

Table 11 provides these details of the density,ρ, specific heat capacity, cp, and the exposed thickness, d. It is assumed that the wall has a plasterboard finish, ceiling is a suspended ceiling system and floor has a carpet/lino finish. Equation 25 shows how the thermal capacitance is calculated for each element

(n):

$$c_n = \rho_n cp_n d_n. \tag{25}$$

These are required to understand the thermal time constant of each building.

Internal gains within the Degree days model must be averaged over 24 hours and this value assumed to be 18.9 W/m^2.

A relatively high occupancy density has been predicted for each space. This leads to area weighted values of 0.36-0.38 $people/m^2$ which is equivalent to that of a classroom [79] where area guidelines shows a typical pupil to floor area ratio of 0.07 - 0.21$pupils/m^2$ [59]. This high occupancy density will be used to estimate ventilation losses due to natural ventilation and has been kept high to make a simplified assumption of the same ventilation patterns throughout the entire school. This also helps account for any scenarios where windows remain open throughout unoccupied hours or the school is used out of hours for different purposes. This value is also used to predict kitchen gas usage and therefore assumes a reasonably wasteful use of gas in each school.

The model is also dependant on defining key monthly data, including the total occupied days, the total heated occupied days and the monthly average temperature difference between incoming cold water and outgoing hot water which are shown in Table 12.

Input	Primary school	Secondary School	College
Set point temperature ($°C$)	19	19	19
Occupancy density* ($person/m^2$)	0.38	0.38	0.38
Occupied hours (hrs)	10	10	10
Internal gains* (W/m^2)	18.9	18.9	18.9
Hot water use* (L/m^2)	0.7	1.9	1.9
Ventilation rate ($L/s.person$)	8	8	8
Kitchen gas usage ($kWh/meal$)	0.41	0.41	0.41
Total occupied days ($days$)	195	195	195
Meal ratio	0.65	0.65	0.65
References: [80, 30, 8, 32, 81, 82]			

Table 10: Inputs for different building typologies within the model.

	Value	Unit	Symbol	Notes	Ref
Wall and ceiling	700	kg/m^3	ρ_w	Plasterboard finish	[8]
	1	$kJ/kg.K$	cp_w		
	0.0125	m	d_w		
Window	2500	kg/m^3	ρ_g	4mm panes	[8]
	0.84	$kJ/kg.K$	cp_g		
	0.004	m	d_g		
Floor	800	kg/m^3	ρ_f	Lino/Carpet finish	[8]
	1.1	$kJ/kg.K$	cp_f		
	0.0085	m	d_f		

Table 11: Properties of the first insulating layer of each internal element.

Month	Occupied days	Heated occupied days	Hot water $\delta\theta$
Jan	23	23	41.2
Feb	15	15	41.4
Mar	13	13	40.1
Apr	12	6	37.6
May	18	0	36.4
Jun	22	0	33.9
Jul	17	0	30.4
Aug	0	0	33.4
Sep	22	0	33.5
Oct	18	9	36.3
Nov	22	22	39.4
Dec	13	13	39.9
References: [35, 30, 82]			

Table 12: Key monthly data, the heating season is assumed to from mid October to mid April.

6.1 Weather data

Key weather data, being monthly average outdoor temperatures and solar gains are provided below in Tables 13 - 14.

Month	EE	EN	M	EA	ES	EC
Jan	4.3	4.7	4.3	4.5	5.2	4.8
Feb	5.7	6	6.5	6.8	7	7.1
Mar	6.9	7	7.4	7.8	7.8	8.1
Apr	8.2	8.1	8.8	9.3	8.9	9.4
May	12.2	11.8	12.8	13.5	12.3	13.3
Jun	14.4	13.6	14.8	15.7	14.2	15.5
Jul	17.6	16.23	18.1	19.3	17.1	19
Aug	17.2	16.5	18.3	19.7	17.9	19.5
Sep	13.7	13.4	14.1	15.1	14.1	14.9
Oct	11.7	11.7	12.4	13.4	12.8	13.6
Nov	8	8.3	8.6	9.4	9.3	9.8
Dec	2.9	3	3.2	3.7	4.1	4.1

Table 13: Average monthly outdoor temperature data ($^{\circ}C$), for the different regions [60]. EE = England East and North East, EN = England North West and Wales North, M = Midlands, EA = East Anglia, ES = England South West and Wales South and EC = England South East and Central.

Solar gains are required to be inputted into the Degree Days equation as a 24 hour monthly average (W/m^2). Solar heat gains in $kWh/m^2/day$ for a North, South, East and West facing wall were found for each month of the year [61]. A 24 hour monthly average of each direction was then found. The assumption that there is an equal window surface on every direction of each building has been made. As described in the main text, a constant window to wall ratio is assumed. This was combined with a typical solar gain factor of 0.62 and a frame factor of 0.3 to account for the fact that not all solar gains hitting window will heat the internal space [33, 83].

Month	EE	EN	M	EA	ES	EC
Jan	20.8	16.9	21.1	22.9	21.1	25.3
Feb	36.3	35.8	38.8	37.9	38.8	36.4
Mar	55	53.1	62.1	55.5	62.1	51.1
Apr	76.4	80.8	84.7	80.7	84.7	77.2
May	92.6	94	99	99.7	99	95.2
Jun	94.8	92.1	104.4	95.8	104.4	89.1
Jul	97.2	85.6	105.8	98.5	105.8	94.1
Aug	85	82	93.3	90	93.3	89
Sep	65.2	67.7	72.2	73.6	72.2	71.1
Oct	36.4	41.5	47.6	50.5	47.6	50.4
Nov	23.2	23.6	26.3	24.9	26.3	30.3
Dec	15.9	14	18.5	15.4	18.5	17.6

Table 14: 24-hour averaged monthly solar irradiance - averaged over North, South, East and West facing walls (W/m^2) [61], for the different Met Office regions [60].

References

[1] "Verisk," OS Digimaps. [Online]. Available: https://digimap.edina.ac.uk/verisk

[2] "Get information about schools," HM Government. [Online]. Available: https://get-information-schools.service.gov.uk/

[3] Ministry of Education, "The story of post-war school building (1957)," Crown Copyright, London, UK, 1957. [Online]. Available: https://www.education-uk.org/documents/minofed/pamphlet-33.html

[4] Department of Education, "Primary schools building handbook," Department of Education, Bangor, UK, Apr 2009.

[5] Department for Education, "Condition of school buildings survey - key findings," Crown Copyright, London, UK, May 2021. [Online]. Available: https://assets.publishing.service.gov.uk/media/60af7cbbe90e071b54214c82/Condition_of_School_Buildings_Survey_CDC1_-_key_findings_report.pdf

[6] M. Jentsch, *Refurbishment of non-domestic buildings - TM53: 2013*, 1st ed., K. Butcher, Ed. CIBSE, 2013.

[7] Department of the Environment, Transport and the Regions, "Energy efficient refurbishment of schools," Crown Copyright, Watford, UK, 1997. [Online]. Available: https://www.greensuffolk.org/app/uploads/2021/05/Energy-efficient-refurbishment-for-schools.pdf

[8] F. Parand, F. Nicol, M. Nikolopoulou, G. Levermore, J. Dixon, M. Eames, V. Hanby, H. Junaidi, T. Muneer, C. Sanders, D. Virk, B. Anderson, M. Liddament, M. Holmes, and D. Arnold, *CIBSE Guide A*, 8th ed., K. Butcher and B. Craig, Eds. CIBSE, 2021.

[9] HM Government, "Conservation of fuel and power volume 2: Approved document l: Buildings other than dwellings," HM Government, London, UK, 2023.

[10] S. Hong, D. Godoy-Shimizu, Y. Schwartz, I. Korolija, A. Mavrogianni, and D. Mumovic, "Characterising the english school stock using a unified national on-site survey and energy database," *Building Services Engineering Research & Technology*, vol. 43, no. 1, pp. 89–112, Jul 2021.

[11] J. Bull, A. Gupta, D. Mumovic, and J. Kimpian, "Life cycle cost and carbon footprint of energy efficient refurbishments to 20th century uk school buildings," *International Journal of Sustainable Built Environment*, vol. 3, no. 1, pp. 1–17, Jun 2014.

[12] J. Harris, *Maintenance engineering and management - CIBSE Guide M*, 2nd ed., B. Craig, Ed. The Chartered Institution of Building Services Engineers, 2014.

[13] SPS Roofing, "How long does a flat roof last?" SPS Roofing, n.d. [Online]. Available: https://spsroofingltd.co.uk/blog/how-long-does-a-flat-roof-last/\#:~:text=However\%2C\%20since\%20the\%20introduction\%20of,you\%20are\%20at\%20a\%20property.

[14] Nottinghamshire County Council, "Timber windows: A sustainable choice," Nottinghamshire County Council, n.d. [Online]. Available: https://www.bassetlaw.gov.uk/media/3573/nccwindowsguide.pdf

[15] Department for Business, Energy and Industrial Strategy, "Household energy efficiency detailed release: Great britain data to december 2020," Department for Business, Energy and Industrial Strategy and National Statistics, London, UK, Mar 2021. [Online]. Available: https://assets.publishing.service.gov.uk/media/6050d729d3bf7f045b9231b1/Detailed_Release_-_HEE_stats_18_Mar_2021_FINAL.pdf

[16] Y. Schwartz, I. Korolija, P. Symonds, D. Godoy-Shimizu, J. Dong, S. M. Hong, A. Mavrogianni, D. Grassie, and D. Mumovic, "Indoor air quality and overheating in uk classrooms - an archetype stock modelling approach," in *Journal of Physics: Conference Series*, vol. 2069, Copenhagen, DenMark, Aug 2021.

[17] Passive House Institute, "Criteria for the passive house, enerphit and phi low energy building standard," Passive House Institute, Darmstadt, Germany, Aug 2016. [Online]. Available: https://passipedia.org/_media/picopen/9f_160815_phi_building_criteria_en.pdf

[18] O. Dura and K. Lomas, "Retrofitting post-war office buildings: Interventions for energy efficiency, improved comfort, productivity and cost reduction," *Journal of Building Engineering*, vol. 42, Oct 2021, Art. no. 102746.

[19] LETI, "Leti climate emergency design guide," LETI, London, UK, Jan 2020. [Online]. Available: https://www.leti.uk/_files/ugd/252d09_3b0f2acf2bb24c019f5ed9173fc5d9f4.pdf

[20] R. Hegarty, O. Kinnane, D. Lennon, and S. Colclough, "Air-to-water heat pumps: Review and analysis of the performance gap between in-use and product rated performance," *Renewable and Sustainable Energy Reviews*, vol. 155, Mar 2022, Art. no. 111887.

[21] R. Lowe, A. Summerfield, E. Oikonomou, J. Love, P. Biddulph, C. G. andL Chiu, and J. Wingfield, "Final report on analysis of heat pump data from the renewable heat premium payment (rhpp) scheme," UCL Energy Institute, Mar 2017. [Online]. Available: https://doc.ukdataservice.ac.uk/doc/8151/mrdoc/pdf/8151_Decc_rhpp_final_report_v1-13.pdf

[22] M. Lammle, C. Bongs, J. Wapler, D. Gunther, S. Hess, M. Kropp, and S. Herkel, "Performance of air and ground source heat pumps retrofitted to radiator heating systems and measures to reduce space heating temperatures in existing buildings," *Energy*, vol. 242, Mar 2022, Art. no. 122952.

[23] National Energy System Operator, "Future energy scenarios - data workbook," NESO, 2023.

[24] Department for Energy Security and Net Zero, "Greenhouse gas reporting: conversion factors 2024," Department for Energy Security and Net Zero, United Kingdom, Oct 2024. [Online]. Available: https://www.gov.uk/government/publications/greenhouse-gas-reporting-conversion-factors-2024

[25] Department for Business, Innovation & Skills and Department of Energy & Climate Change, "Industrial decarbonisation and energy efficiency roadmaps to 2050," Department for Business, Innovation & Skills and Department of Energy & Climate Change, Mar 2015. [Online]. Available: https://www.gov.uk/government/publications/industrial-Decarbonisation-and-energy-efficiency-roadmaps-to-2050

[26] PEP eco Passport, "Pep eco passport," 2024. [Online]. Available: https://register.pep-ecopassport.org/

[27] E. Aydin, N. Kok, and D. Brounen, “Energy efficiency and household behavior: the rebound effect in the residential sector,” *The RAND Journal of Economics*, vol. 48, no. 3, pp. 749–782, Aug 2017.

[28] S. Pauliuk, K. Sjöstrand, and D. B. Müller, “Transforming the norwegian dwelling stock to reach the 2 degrees celsius climate target,” *Journal of Industrial Ecology*, vol. 17, no. 4, pp. 542–554, Jan 2013.

[29] National Education Union, “Class sizes,” Website, National Education Union, London, UK, Oct 2024. [Online]. Available: https://neu.org.uk/advice/classroom/class-sizes

[30] Energy sparks, “Switched off heating for the summer,” Website, Energy sparks, n.d. [Online]. Available: https://energysparks.uk/intervention_types/31_

[31] Energy Saving Trust, “Measurement of domestic hot water consumption in dwellings,” Defra, 2008. [Online]. Available: https://assets.publishing.service.gov.uk/media/5a75a29ced915d6faf2b4829/3147-measure-domestic-hot-water-consump.pdf

[32] R. Burgiss and L. Rose, *Energy efficiency in commercial kitchens - TM51:2021*. CIBSE, 2021.

[33] T. Day, *Degree-days: theory and application TM41: 2006*, 1st ed. CIBSE, 2006.

[34] BRE for the Department for Communities and Local Government (DCLG), “A technical manual for sbem part of the national calculation methodology : Sbem for assessing the energy performance of buildings,” the Department for Communities and Local Government (DCLG), Nov 2015.

[35] Building Research Establishment Ltd (BRE), “Amendments to sap’s hot water methodology,” Building Research Establishment Ltd (BRE), Jul 2016. [Online]. Available: https://bregroup.com/documents/d/bre-group/consp-08-hot-water-v1_0-pdf

[36] “Energy performance of buildings data: England and wales,” Department for Levelling up and Housing. [Online]. Available: https://epc.opendatacommunities.org/

[37] J. Field, *Energy benchmarks CIBSE TM46:2008*. The Chartered Institution of Building Services Engineers, 2008.

[38] P. Schild and M. Mysen, “Technical note aivc 65 - recommendations on specific fan power and fan system efficiency,” Air Infiltration and Ventilation Centre, Lozenberg, Belgium, Dec 2009. [Online]. Available: https://www.aivc.org/sites/default/files/members_area/medias/pdf/Technotes/TN65_Specific\%20Fan\%20Power.pdf

[39] British Standards Institute, *BS EN 15643:2021:Sustainability of construction works. Framework for assessment of buildings and civil engineering works.* BSI, 2022.

[40] G. Grazieschi, F. Asdrubali, and G. Thomas, "Embodied energy and carbon of building insulating materials: A critical review," *Cleaner Environmental Systems*, vol. 2, Jun 2021, Art. no. 100032.

[41] Knauf Insulation, "Environmental product declaration," 2020. [Online]. Available: https://www.knaufinsulation.si/sites/ki_si/files/images/BW-Supafil_Loft_FINAL.pdf

[42] ——, "Environmental product declaration - venti pro," 2021. [Online]. Available: https://pim.knaufinsulation.com/files/download/rmw_pf_a2_s-p-03705.pdf

[43] DANOSA, "Environmental product declaration - danopol pvc waterproofing sheet," 2021. [Online]. Available: https://api.environdec.com/api/v1/EPDLibrary/Files/ec553b69-9c22-4557-8222-08d941d5f1c9/Data

[44] Nordia, "Environmental product declaration - cement-based floor screeds by nordia s.a," 2023. [Online]. Available: https://api.environDec.com/api/v1/EPDLibrary/Files/4d2f5a6a-fe83-43c5-f702-08dbfcbc4c1b/Data

[45] Partel, "Environmental product declaration - vapour control membranes," 2022. [Online]. Available: https://www.igbc.ie/wp-content/uploads/2022/07/EPD-Partel-Vapour-Control-Membranes-18-07-2022-EPDIE-22-83.pdf

[46] ISOPLAC, "Environmental product declaration - isoplac plasterboards," 2022. [Online]. Available: https://api.environDec.com/api/v1/EPDLibrary/Files/ebcc6915-f5ca-48fa-c24a-08dab10e0dce/Data

[47] UPMBioFore, "Environmental product declaration - standard and special sawn timber," 2022. [Online]. Available: https://api.environDec.com/api/v1/EPDLibrary/Files/c900df79-5c86-4797-b097-08daac2bcf89/Data

[48] Knauf Insulation, "Environmental product declaration - omnifit slab 35," 2023. [Online]. Available: https://api.environDec.com/api/v1/EPDLibrary/Files/d19618e9-6a5a-4b89-16bd-08dbca69748b/Data

[49] Munster Joinery, "Passiv pvc double glazed window," 2018. [Online]. Available: https://www.igbc.ie/wp-content/uploads/2018/12/EPD-MunsterJoinery-689-16.11.18-EPDIE-18-09-1.pdf

[50] ——, "Passiv pvc triple glazed window," 2018. [Online]. Available: https://www.igbc.ie/wp-content/uploads/2018/06/EPD-MunsterJoinery-EPDIE-18-08.pdf

[51] Mitsubishi, "i-fx-n-go5/sl-a 1152 - cibse tm65 embodied carbon mid-level calculation," 2022. [Online]. Available: https://library.mitsubishielectric.co.uk/pdf/book/i-FX-N-1152-TM65-Embodied-Carbon-Calculation\#page-1

[52] ——, "Qahv-n560ya-hpb - cibse tm65 embodied carbon mid-level calculation," 2021. [Online]. Available: https://library.mitsubishielectric.co.uk/pdf/book/QAHV-N560YA-HPB-TM65-Calculation\#page-1

[53] Polypipe, "Environmental product declaration - mecflow and terrain q systems," 2022. [Online]. Available: https://api.environDec.com/api/v1/EPDLibrary/Files/cec21caa-9662-4d2d-cdce-08d9df0ea78f/Data

[54] CHAPPÉE, "Samba classique / 6t / collectivité - horizontal," CHAPPÉE, Sep 2019. [Online]. Available: https://register.pep-ecopassport.org/pep/consult/mbesqrsCBZbWbKJq6-kJ3m2JfBkDSgTolqQnfAUXdbE/mbesqrsCBZbWbKJq6-kJ3nXTEwaL2H-VUQApFU2-Q6g

[55] ——, "Samba pure verticall," CHAPPÉE, Sep 2019. [Online]. Available: https://register.pep-ecopassport.org/pep/consult/mbesqrsCBZbWbKJq6-kJ3hGcl2CRBEji7MXjXFTkvmg/mbesqrsCBZbWbKJq6-kJ3nXTEwaL2H-VUQApFU2-Q6g

[56] VELTEK Ventilation, "Verified environmental product declaration," 2023. [Online]. Available: https://www.epddanMark.dk/media/oe1b3bgs/md-23023-en.pdf

[57] Wildeboer Bauteile GmbH, "Eckige volumenstromregler vke und vk -wildeboer bauteile gmbh," 2021. [Online]. Available: https://ibudata.lca-data.com/resource/sources/b6b609c3-3f29-4ae5-af65-4e4de9c2756a/Volumenstromregler_VKE_VK_14509.pdf?version=00.01.000

[58] C. Parsloe and M. Ratcliffe, *Heating CIBSE Guide B1:2016*, 1st ed., K. Butcher, Ed. CIBSE, 2016.

[59] Department of Education, "Area guidelines for mainstream schools," Crown Copyright, Jun 2014. [Online]. Available: https://assets.publishing.service.gov.uk/government/uploads/system/uploads/attachment_data/file/905692/BB103_Area_Guidelines_for_Mainstream_Schools.pdf

[60] Metoffice, "Uk and regional series," Metoffice, n.d. [Online]. Available: https://www.metoffice.gov.uk/research/climate/maps-and-data/uk-and-regional-series

[61] National Renewable Energy Laboratory, "Pvwatts calculator," National Renewable Energy Laboratory, n.d. [Online]. Available: https://pvwatts.nrel.gov/index.php

[62] X. Li, H. Arbabi, G. Bennett, T. Oreszczyn, and D. D. Tingley, "Net zero by 2050: Investigating carbon-budget compliant retrofit measures for the english housing stock," *Renewable and Sustainable Energy Reviews*, vol. 161, Jun 2022, Art. no. 112384.

[63] Committee on Climate Change, "The sixth carbon budget - the uk's path to net zero," Committee on Climate Change, London, UK, Dec 2020. [Online]. Available: https://www.theccc.org.uk/wp-content/uploads/2020/12/The-Sixth-Carbon-Budget-The-UKs-path-to-Net-Zero.pdf

[64] J. Kuriakose, C. Jones, K. Anderson, J. Broderick, and C. McLachlan, "The tyndall carbon budget tool," University of Manchester, 2024. [Online]. Available: https://carbonbudget.manchester.ac.uk/reports/

[65] UK Green Building Council, "Net zero whole life carbon roadmap," UK Green Building Council, London, UK, 2021. [Online]. Available: https://www.ukgbc.org/wp-content/uploads/2021/11/UKGBC-Whole-Life-Carbon-Roadmap-A-Pathway-to-Net-Zero.pdf

[66] Department of Education, "Policy paper sustainability and climate change: a strategy for the education and children's services systems," Department of Education, 2023. [Online]. Available: https://www.gov.uk/government/publications/sustainability-and-climate-change-strategy/9317e6ed-6c80-4eb9-be6d-3fcb1f232f3a\#:~:text=Leading\%20by\%20example\%3A\%20cutting\%20energy,are\%20also\%20significant\%20and\%20rising.

[67] Committee on Climate Change, "The sixth carbon budget - buildings," Committee on Climate Change, London, UK, 2020. [Online]. Available: https://www.theccc.org.uk/wp-content/uploads/2020/12/Sector-sumMary-Buildings.pdf

[68] P. Morrell and A. Day, "Testing for a safer future - an independent review of the construction products testing regime," Department for Levelling Up, Housing and Communities, London, UK, Apr 2023. [Online]. Available: https://assets.publishing.service.gov.uk/media/6440f2596dda69000d11e15e/Independent_Review_of_the_Construction_Product_Testing_Regime.pdf

[69] Department for Business and Trade, "Construction building materials: commentary may 2024," Department for Business and Trade, Jun 2024. [Online]. Available: https://www.gov.uk/government/statistics/building-materials-and-components-statistics-May-2024/construction-building-materials-commentary-May-2024

[70] HESA, "Buildings and spaces by he provider and academic year," HESA, Oct 2024. [Online]. Available: https://www.hesa.ac.uk/data-and-analysis/estates/table-1

[71] Writtle University College, “Report and financial statements year ended 31 july 2019,” Writtle University College, Chelmsford, UK, 2019. [Online]. Available: https://writtle.ac.uk/pdfs/5/Report-and-Financial-Statement-2019.pdf

[72] SOAS, “Estate strategy - 2023 - 2028,” SOAS, Nov 2022. [Online]. Available: https://www.soas.ac.uk/sites/default/files/2023-06/Estates-Strategy-2023-2028.pdf

[73] Ravensbourne University London, “Our building,” Website, Ravensbourne University London, n.d. [Online]. Available: https://www.ravensbourne.ac.uk/student-life/our-building\#:~:text=Featuring\%20a\%20variety\%20of\%20workspaces,disciplinary\%2C\%20open\%2Dplan\%20work\%20spaces

[74] Edge Hill University, “Facilities managment,” Website, Edge Hill University, n.d. [Online]. Available: https://www.edgehill.ac.uk/departments/support/fm/

[75] Arts University Plymouth, “About us,” Website, Arts University Plymouth, n.d. [Online]. Available: https://www.aup.ac.uk/about-us/our-campus

[76] RIBA, “Riba 2030 climate challenge,” RIBA, London, UK, 2021.

[77] LETI, “Embodied carbon target alignment,” LETI, n.d. [Online]. Available: https://www.leti.uk/_files/ugd/252d09_25fc266f7fe44a24b55cce95a92a3878.pdf

[78] B. D’Amico and F. Pomponi, “A compactness measure of sustainable building forms,” *Royal Society Open Science*, vol. 6, no. 6, Jun 2019.

[79] M. Mysen, S. Bernsten, P. Nafstad, and P. Schild, “Occupancy density and benefits of demand-controlled ventilation in norwegian primary schools,” *Energy and Buildings*, vol. 37, no. 12, pp. 1234–1240, Dec 2005.

[80] VES, “Technical guide - building bulleting 101 update,” VES, Hampshire, UK, Feb 2019. [Online]. Available: https://ves.co.uk/uploads/docs/bb101-ventilation-schools-technical-guide-2019.pdf

[81] ParentPay, Cypad, and LACA, “2022 school meals report,” ParentPay and Cypad and LACA, 2022. [Online]. Available: https://www.laca.co.uk/sites/default/files/attachment/news/ParentPay\%20School\%20Meal\%20Report\%2006.22\%20(JON).pdf

[82] R. Long, “The school day and year,” House of Commons Library, Aug 2023. [Online]. Available: https://researchbriefings.files.parliament.uk/documents/SN07148/SN07148.pdf

[83] S. Saadatian, F. Friere, and N. Simoes, "Embodied impacts of window systems: A comparative assessment of framing and glazing alternatives," *Journal of Building Engineering*, vol. 35, Mar 2021, Art. no. 102042.
