# Supplementary material for: Actions to decarbonize English schools: a whole life carbon stock assessment
Source: J Ind Ecol. 2026 Feb 26;30(1):391–403. doi: 10.1007/s44498-026-00027-x (PMC13152907; doi:10.1007/s44498-026-00027-x)
Supplement: Supplementary file 2 — (PDF 737 KB) [file 44498_2026_27_MOESM2_ESM.pdf]

Supplementary Information–Results

Actions to decarbonize English schools: A whole life carbon stock assessment

Danielle Abbey*, Hadi Arbabi*, and Danielle Densley Tingley*†

November 18, 2025

This Supplementary Information provides further details and discussion for the results presented within this paper. The SI addresses validation efforts against past work and benchmark data, influence of building form on operational and whole life carbon, and a brief look at sensitivity to occupancy assumptions.

keywords: *industrial ecology; whole life carbon; retrofit; carbon budget; decarbonisation pathways; building material*

*School of Mechanical, Aerospace, & Civil Engineering, University of Sheffield, UK
†d.densleytingley@sheffield.ac.uk

1 Validation against past work and benchmark data

This section compares key results against current available data and benchmarks.

1.1 Total floor area

Search radii were used to define all school buildings based on typical area breakdowns [1] - 85m for Primary, 175m for Secondary and 85m for colleges. To ensure that this study does not miss out on large amounts of data, the total floor area of data collected is compared to the floor area of schools as attained by the school condition survey. The area collected by this survey is defined as *measured off Ordnance Survey (OS) [?] based information using the external face of the perimeter walls at each floor level, and which also includes areas such as those occupied by internal walls and partitions* [2]. Therefore, this can be compared directly to the building area data collected in this paper.

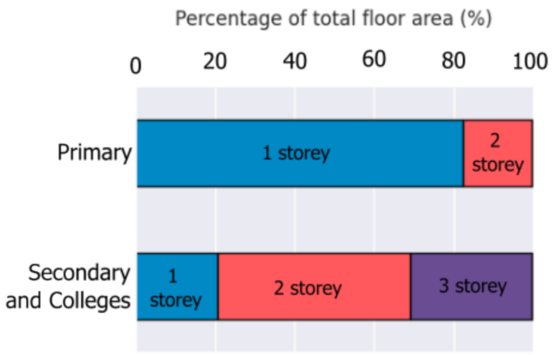

Figure 1: Bar chart to show the distribution of number of storeys within the 235 case study schools.

Interestingly, while primary school database underestimates the **number of properties** by 9%, secondary schools and colleges overestimate by 5%. This perhaps indicates some wrong categorisation within the *Get information about schools* database. This leads to a total of 18,702 schools compared to the 20,109 stipulated by the school condition survey [2].

Within the methods section, it is outlined that a distribution of typical number of storeys is applied to the database, based on Figure 1. For example, approximately 20% of the total primary school floor area within Figure 1 are 2 storey buildings. Therefore the same proportion of the total floor area within the database are classed as two storeys.

Adopting this method leads to a 7% overestimation of floor area for secondary and a 0% overestimation of floor area for primary schools. This implies

that assumptions made about the number of storeys helps account for any missing schools within the database.

Also, **considering the school condition dataset allows for a** $\pm 10\%$ **tolerance in total floor area these values for both secondary and primary are deemed acceptable** within the model with an acknowledgement that they may likely lead to a slight overestimation of carbon emissions due to increased floor area of secondary schools.

It should be noted that a total of 18702 schools were assessed in this study, which is lower than the predicted value of 20,109 properties. However when building floor area is calculated, the total floor area of all schools is within 5% of the School Condition Surveys predicted total floor area. Possible reasons for this include a slight overestimation of the number of storeys for each school or that multiple schools have been merged into one property.

1.2 Total energy consumption

Table 1 shows the final total energy load, including all thermal and electrical consumption, for the PartL2B and Heat Pump only scenario. The results range between $5.05 - 5.8TWh$ which is in line with UKGBC predicted 2050 energy load, stating a total educational stock load of $10TWh$ [3]. Primary, secondary schools and colleges take up approximately 55% of total carbon weighted floor area (See Supplementary Information–Methods) and England accounts for 81 and 85% of UK current electricity and gas usage respectively [4, 5]. Therefore, $5.05 - 5.8TWh$ is similar to the UKGBC value of approximately $4.5 - 4.6TWh$ for this stock type. Results were not expected to be exactly the same as the UKGBC retrofit modelling also includes improved lighting and controls which could help explain the slightly smaller predicted number [3].

	**PartL2B**	**Heat Pump only**
**Final total yearly energy load (*TWh*)**	5.05	5.83

Table 1: Total yearly estimated load in 2050 for the English educational stock post retrofit. **Assuming all stock has been retrofit by 2050.**

1.3 Total embodied carbon

The modelled upfront embodied carbon for PartL2B is equivalent to $73KgCo2e/m^2$ and $50KgCo2e/m^2$ for Falling short and Leading the way respectively. This model is therefore in line with existing embodied carbon benchmarking, where 36 energetic refurbishment projects were found to have an upfront embodied carbon ranging between $20 - 140KgCo2e/m^2$ [6].

The benchmark carbon payback period of up to 8 years for commercial real estate for medium or deep refurbishment [6] is in line with the study findings of a carbon payback period of more than 7 years for older and 11 years for newer buildings. Especially considering that the 8 year calculation only accounts for upfront (A1 - A3) emissions. Validation against past work and benchmark data

2 Influence of building form

One key quality of a building that cannot be changed easily through retrofit is building form. Form aims to measure efficiency in which the external envelope encloses the internal volume of a space. Therefore measures of building form can be used to understand the level of efficiency of a building's shape and size. **An explanation of the measures used in this paper to understand building form are provided in the Supplementary Information–Methods**.

Figures 2b - 2c shows the distribution of building forms for the entire school stock compared to those whose whole life carbon is larger than the LETI A+ band, $260 kgCO_2e/m^2$, by 2050 [7]. The whole life carbon is measured from 2025 - 2050 using the no decarbonisation scenario.

Figures 2b - 2c show that the distribution of forms in Figure 2c have a tendency for a lower aspect ratio ($r = \frac{W}{L}$) combined with a higher slenderness ($k = \frac{H}{L}$) compared to the distribution for the whole stock. The distribution of forms for the whole stock, as in Figure 2b, is shown to cluster much more heavily toward a more efficient form, with low slenderness values.

The five highest whole life carbon values [$kgCO_2e/m^2$] are shown to all have small plan areas. This is because as one reduces aspect ratio and increase slenderness the range of realistic building forms become limited. The range in slenderness values is especially limited as the archetypal number of storeys, x, ranges between 1 and 3 for this stock type. At smaller plan areas a single or double storey building can still have a reasonably high slenderness and is more likely to have this combined with a very low aspect ratio. Therefore, even if LETI Band A+, $260 kgCO_2e/m^2$, was achieved and these buildings replaced it would likely have a small impact on the total floor area of our school stock.

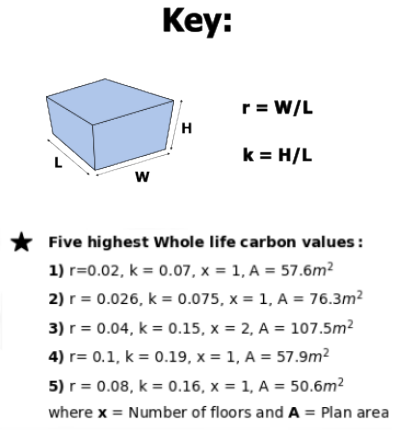

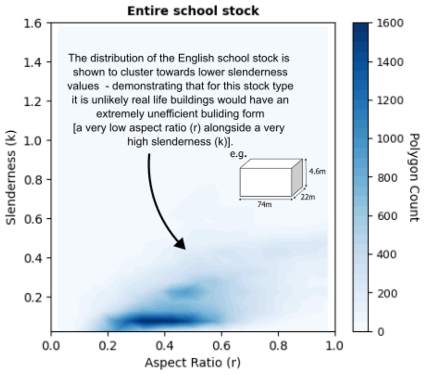

(b) Distribution of the all English school stock.

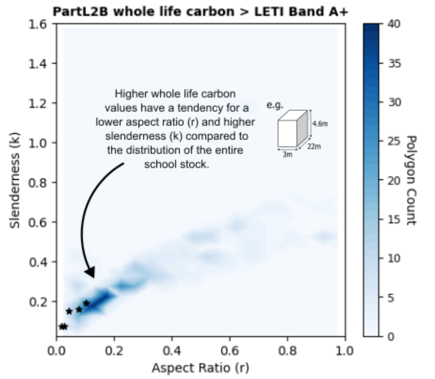

(c) Distribution of those polygons with a whole life carbon higher than LETI Band A+.

Figure 2: The distribution of building forms for the whole stock as well as those whose whole life carbon is higher than LETI Band A+, $260kgCO_2e/m^2$, by 2050, assuming no decarbonisation. Darker colours insinuate a higher polygon count.

3 Occupancy Sensitivity analysis

The minimum, typical, and maximum post retrofit occupancy schedules have been modelled for all the key results. These are demonstrated in Figures 3a - 3l.

Figures 3a - 3l show that reducing occupancy usage, e.g. heat set point temperature, total heated days and hot water consumption, reduces overall carbon emissions and increasing usage, the opposite. Decarbonisation scenarios with higher total emissions, e.g. no decarbonisation, have a larger difference

between different occupancy patterns due to the higher proportion of emissions which come from operational energy consumption.

The key finding is that overall patterns and conclusions drawn do not change between these different sensitivities. For example, the CCC carbon budget cannot be met without, multi-sector decarbonisation as well as a marked increase in retrofit rates compared to current practise. This retrofit rate is also still benefited by prioritisation of least thermally efficient buildings. The Tyndall budget can not be met once you include considerations of upfront and replacement carbon emissions.

Perhaps the most significant finding is the marked increase in retrofit rates due to a potential increase in post-retrofit occupancy usage. In fact, the required rate would nearly double under the Falling short decarbonisation scenario. As the rebound effect has been evidenced within past work [8], the potential for an increase in post retrofit energy consumption is shown to pose a very real threat for the ability to meet the CCC budget. This should be accounted for in future retrofit decisions, with either an increase in retrofit rates if this is expected to occur or measures to help reduce the likelihood of occurrence such as simple and easy to use building control systems.

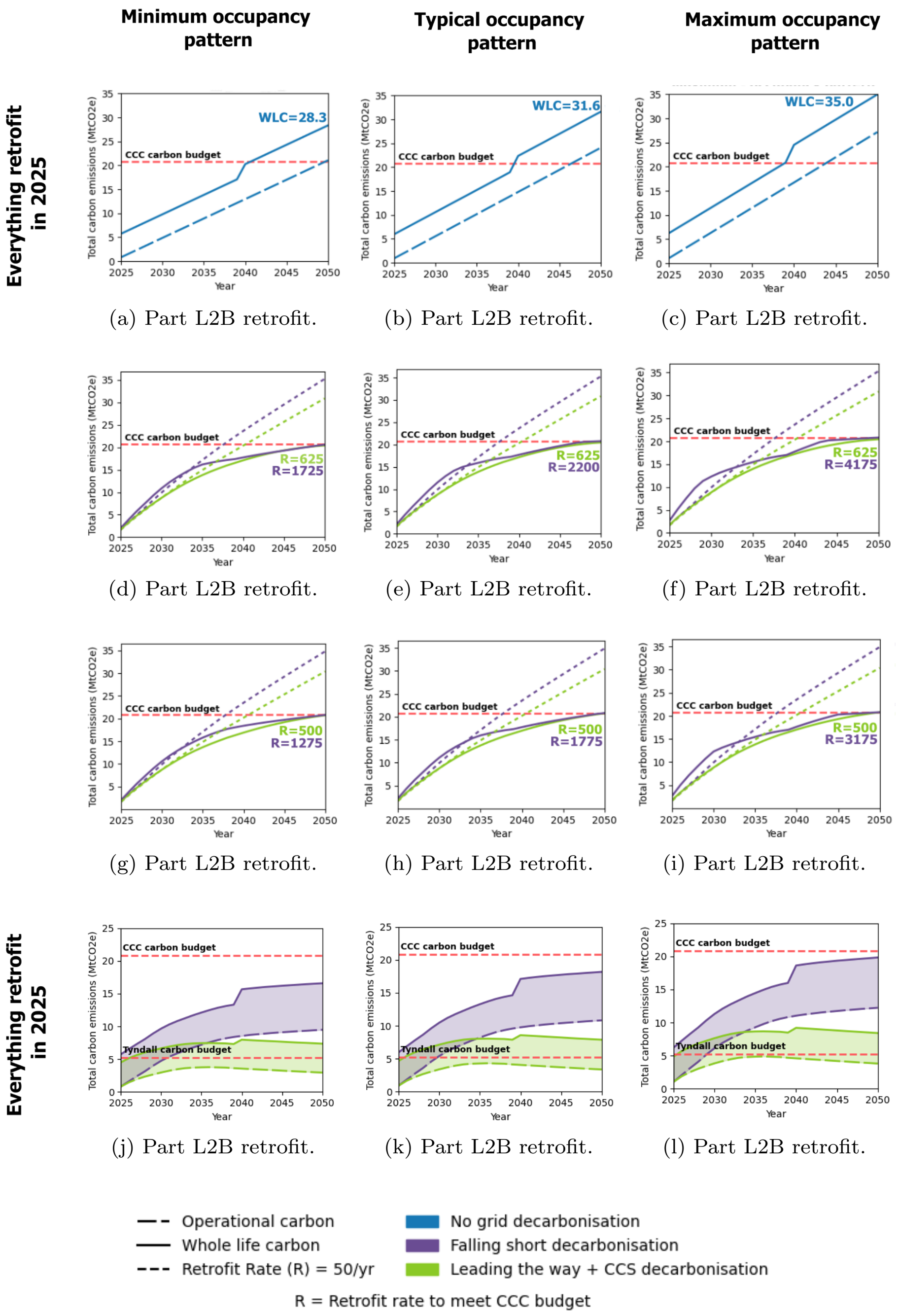

(a) Part L2B retrofit. (b) Part L2B retrofit. (c) Part L2B retrofit.

(d) Part L2B retrofit. (e) Part L2B retrofit. (f) Part L2B retrofit.

(g) Part L2B retrofit. (h) Part L2B retrofit. (i) Part L2B retrofit.

(j) Part L2B retrofit. (k) Part L2B retrofit. (l) Part L2B retrofit.

Figure 3: Sensitivity analysis to show the potential impact of changes to occupancy schedules - post retrofit.

References

[1] "Get information about schools," HM Government. [Online]. Available: https://get-information-schools.service.gov.uk/

[2] Department for Education, "Condition of school buildings survey - key findings," Crown Copyright, London, UK, May 2021. [Online]. Available: https://assets.publishing.service.gov.uk/media/60af7cbbe90e071b54214c82/Condition_of_School_Buildings_Survey_CDC1_-_key_findings_report.pdf

[3] UK Green Building Council, "Net zero whole life carbon roadmap," UK Green Building Council, London, UK, 2021. [Online]. Available: https://www.ukgbc.org/wp-content/uploads/2021/11/UKGBC-Whole-Life-Carbon-Roadmap-A-Pathway-to-Net-Zero.pdf

[4] M. Gregory, S. Khan, and S. Stadnyk, "Sub-national gas consumption statistics," Department of Energy and Climate Change, 2013. [Online]. Available: https://assets.publishing.service.gov.uk/media/5a7f094240f0b62305b84b7f/sub_national_gas_consumption_factsheet_2012.pdf

[5] H. Clark and T. Roberts, "Electricity generation and supply figures for scotland, wales, northern ireland and england, 2014 to 2017," Department for Energy Security and Net Zero and Department for Business, Energy and Industrial Strategy, n.d. [Online]. Available: https://assets.publishing.service.gov.uk/media/5c3c688d40f0b67c62695b27/Regional_Electricity_Generation_and_Supply.pdf

[6] S. Bienert, H. Kuhlwein, Y. Schmidt, B. GLoria, and B. Agbayir, "Embodied carbon of retrofits. ensuring the ecological payback of energetic retrofits," IIO and CRREM initiative, Worgl, Austria, Sep 2023. [Online]. Available: https://www.crrem.eu/wp-content/uploads/2023/09/Report-Embodied-carbon-vs-operational-savings_Sep23.pdf

[7] LETI, "Embodied carbon target alignment," LETI, n.d. [Online]. Available: https://www.leti.uk/_files/ugd/252d09_25fc266f7fe44a24b55cce95a92a3878.pdf

[8] E. Aydin, N. Kok, and D. Brounen, "Energy efficiency and household behavior: the rebound effect in the residential sector," *The RAND Journal of Economics*, vol. 48, no. 3, pp. 749–782, Aug 2017.
